# Laboratory and Numerical Investigation of Pre-Tensioned Reinforced Concrete Railway Sleepers Combined with Plastic Fiber Reinforcement

**DOI:** 10.3390/polym16111498

**Published:** 2024-05-24

**Authors:** Attila Németh, Sarah Khaleel Ibrahim, Majid Movahedi Rad, Szabolcs Szalai, Zoltán Major, Szabolcs Kocsis Szürke, Vivien Jóvér, Mykola Sysyn, Dmytro Kurhan, Dániel Harrach, Gusztáv Baranyai, Imre Fekete, Richárd Nagy, Hanna Csótár, Klaudia Madarász, András Pollák, Bálint Molnár, Bence Hermán, Miklós Kuczmann, László Gáspár, Szabolcs Fischer

**Affiliations:** 1 Central Campus Győr, Széchenyi István University, H-9026 Győr, Hungary; nemeth.attila@sze.hu (A.N.); sarah.khaleel.ibrahim@hallgato.sze.hu (S.K.I.); majidmr@sze.hu (M.M.R.); szalaisz@sze.hu (S.S.); majorz@sze.hu (Z.M.); kocsis.szabolcs@ga.sze.hu (S.K.S.); jover.vivien@sze.hu (V.J.); harrach.daniel@sze.hu (D.H.); baranyai.gusztav@sze.hu (G.B.); fekete.imre@sze.hu (I.F.); nagy.richard@sze.hu (R.N.); csotar.hanna@sze.hu (H.C.); madarasz.klaudia@sze.hu (K.M.); pollak.andras@sze.hu (A.P.); molnar.balint@sze.hu (B.M.); herman.bence@sze.hu (B.H.); kuczmann@sze.hu (M.K.); 2 Vehicle Industry Research Center, Széchenyi István University, H-9026 Győr, Hungary; gaspar.laszlo@kti.hu; 3 Department of Planning and Design of Railway Infrastructure, Technical University Dresden, D-01069 Dresden, Germany; mykola.sysyn@tu-dresden.de; 4 Department of Transport Infrastructure, Ukrainian State University of Science and Technologies, UA-49005 Dnipro, Ukraine; d.m.kurhan@ust.edu.ua; 5 KTI Institute for Transport Sciences Non-Profit Ltd., H-1119 Budapest, Hungary

**Keywords:** railway, reinforced concrete, sleeper, pre-stressing, plastic fiber reinforcement, laboratory experiments, numerical modeling, FEM, ABAQUS, DIC

## Abstract

This research investigates the application of plastic fiber reinforcement in pre-tensioned reinforced concrete railway sleepers, conducting an in-depth examination in both experimental and computational aspects. Utilizing 3-point bending tests and the GOM ARAMIS system for Digital Image Correlation, this study meticulously evaluates the structural responses and crack development in conventional and plastic fiber-reinforced sleepers under varying bending moments. Complementing these tests, the investigation employs ABAQUS’ advanced finite element modeling to enhance the analysis, ensuring precise calibration and validation of the numerical models. This dual approach comprehensively explains the mechanical behavior differences and stresses within the examined structures. The incorporation of plastic fibers not only demonstrates a significant improvement in mechanical strength and crack resistance but paves the way for advancements in railway sleeper technology. By shedding light on the enhanced durability and performance of reinforced concrete structures, this study makes a significant contribution to civil engineering materials science, highlighting the potential for innovative material applications in the construction industry.

## 1. Introduction

### 1.1. General Introduction

Transportation has been crucial in driving growth and development throughout human history [1,2]. The development of the wheel, advances in marine transportation, the Industrial Revolution with its breakthrough steam engine technology, and later inventions, such as electricity and internal combustion engines, are all key milestones that represent substantial improvements [3,4]. Today, transport sciences comprise a wide range of disciplines, such as transport engineering, logistics and transport packing, civil engineering, electrical engineering, mechanical engineering, vehicle engineering, computer science, and economics [5,6,7,8].

It is critical to address three primary transportation disciplines: land transportation, air transportation (including space travel), and water transportation. These disciplines interact and play important roles in many subdisciplines of transportation. On the other hand, pipeline transport functions differently and is primarily concerned with the flow of gas and oil rather than passenger transit. This is why pipeline transport is not addressed in this context.

In each of these fields, significant considerations such as sustainability, ecological operation, and economics must be considered [9,10,11].

This article will examine rail transportation, especially focusing on railway sleepers, and will pay dedicated attention to concrete structures reinforced by plastic fibers (see Section 1.2).

Before describing the particular field of the article, the authors would like to discuss general topics as introductory themes. The paper is mainly related to civil engineering; however, other engineering disciplines (e.g., mechanical engineering, material engineering, and technologies) are also being brought as appropriate and connecting fields.

In the case of traditional ballasted railway permanent ways, their structure consists of the superstructure, i.e., rails, sleepers, rail fasteners (fastening system), ballast bed with its subballast, and the substructure, i.e., granular protection layer (formation layer or protection layer) and the subgrade [12,13,14,15,16]. In European terminology, subballast refers to the lower layer of the ballast bed beneath the sleepers; in American terminology, however, subballast is synonymous with the formation layer and is classified as part of the substructure. The elements of the layer structure constitute a (force) support system in which vertical and horizontal loads (forces) are distributed according to a given distribution law.

### 1.2. Railway Sleepers

Railway sleepers, which are crucial for constructing and maintaining railway tracks, provide essential support and stability for the safe operation of trains [17,18]. Historically, materials like wood, concrete (mainly reinforced concrete—i.e., RC), steel, and synthetics have offered unique benefits [17,18]. Reinforced concrete sleepers can increase the load-bearing capacity of structures, simultaneously decreasing deformation due to loading and the crack width in the concrete [19]. The railway companies’ experiences with these reinforced structures are favorable [20].

The current paper deals with pre-stressed reinforced concrete railway sleepers with and without additional plastic fiber reinforcement. Concrete sleepers are favored for their durability and low maintenance and are particularly suitable for high-speed rails. Reinforced with steel rods or fibers, reinforced concrete sleepers offer improved strength and resilience and are ideal for areas with heavy loads and temperature extremes. Fiber-reinforced sleepers combine the benefits of reinforced concrete with additional strength and maintenance efficiency, making them suitable for high-stress areas.

Adding extra fiber reinforcement to steel-reinforced concrete sleepers represents a major advancement in railway infrastructure. This review compiles findings from various studies, highlighting how fiber reinforcement affects the performance of concrete sleepers. Each statement reflects the organized content from the provided dataset to ensure precision and thoroughness.

The studies investigated different materials for sleepers, including recycled chopped carbon fiber and epoxy resin [21], pre-stressed concrete with CFRP (Carbon Fiber-Reinforced Polymer) [22], and synthetic fiber-reinforced concrete [23]. Other studies looked at materials like laminated form carbon [24] and steel-reinforced concrete [25]. These varied materials underscore the broad exploration of fiber reinforcement in enhancing sleeper properties.

The fibers used in these studies included carbon fibers, synthetic fibers, and hybrid mixes of steel and polypropylene fibers [21,22,23,25]. Carbon fibers are popular for their high strength and stiffness [21]. The quantity of fibers varied, with some studies optimizing the content to 2 wt% carbon fiber for the best results [21], while others tested amounts like 2.0 kg/m^3^ and 5.0 kg/m^3^ [23]. This variation was crucial in identifying the most effective reinforcement levels.

The main methodologies involved lab experiments to evaluate the mechanical and dynamic properties of sleepers. These included flexural strength tests, rail seat static positive moment tests, and dynamic damping assessments [21,22,23,24,25]. The studies aimed to reduce vibration and noise issues [21], compare modal and harmonic responses [22], and assess flexural tensile strength [23]. For instance, tests on CFRPC sleepers aimed to mitigate vibration and noise problems [21].

Comparative studies were vital, contrasting fiber-reinforced sleepers with traditional steel-reinforced ones. The results consistently showed that fiber reinforcement significantly enhanced the mechanical properties of concrete sleepers. CFRPC sleepers, for example, demonstrated better flexural strength and reduced vibration compared to conventional sleepers [21]. Similarly, CFRP-reinforced, non-pre-stressed sleepers performed similarly to pre-stressed B70-type sleepers [22]. Adding synthetic fibers to concrete greatly improved its mechanical properties, like flexural tensile strength [23].

Incorporating additional fibers into concrete sleepers provided many benefits. Enhanced durability and strength were common themes [21,22,23,25]. Adding fibers like synthetic and carbon fibers increased resistance to cracking and improved load-bearing capacity [23]. Polypropylene fibers, for example, contribute to better energy absorption and distribution during impact, which is crucial for the longevity and safety of railway infrastructure [24]. Another study found that LCR-6 sleepers with additional fiber reinforcement performed better than conventional sleepers [25].

Despite these benefits, challenges with fiber reinforcement remain. One major issue is the complexity of manufacturing, which could increase production costs [23]. Ensuring even the distribution of fibers within the concrete matrix is critical for consistent performance, requiring precise mixing and placement techniques [22]. Additionally, the long-term performance and environmental impact of synthetic fibers need further study [24]. The main conclusions from the study on LCR-6 sleepers indicated that adding fibers significantly improved performance, although manufacturing complexities were noted [25].

Table 1 contains the results of a very detailed literature review with more considered aspects [21,22,23,24,25,26,27,28,29,30,31,32,33,34,35,36,37,38,39,40,41,42,43,44,45,46,47,48,49].

### 1.3. Identification of the Research Gap and the Structure of the Current Paper

The review of the current literature highlights a notable gap in the study of plastic fiber reinforcement in (pre-stressed) steel-reinforced concrete railway sleepers (see Section 1.1 and Section 1.2). While many studies have explored high-strength fibers like carbon and polypropylene, the potential benefits of plastic fibers have been largely overlooked. There is also a lack of comprehensive comparisons and long-term performance data for sleepers reinforced with plastic fibers. This research aims to address these gaps by performing thorough laboratory tests and numerical modeling to assess the mechanical properties and overall performance of plastic fiber-reinforced sleepers. By doing so, it seeks to demonstrate the feasibility, cost-effectiveness, and durability of using plastic fibers in railway sleeper construction.

The current paper is the so-called continuation of the authors’ previous publication [19]. The main aim was to investigate a unique plastic fiber-reinforced pre-stressed concrete railway sleeper in the laboratory and in sophisticated finite element (FE) software, namely ABAQUS. The entire examination is founded on the procedure in which the behavior of the above-mentioned unique sleeper is compared with the same product without plastic fiber reinforcement. It should be mentioned that the basis for pre-stressed reinforced-concrete sleepers were the L4-type standard sleepers manufactured by the MABA Hungária Ltd. (Hungary) in Várpalota [50,51] the unique sleepers received additional plastic fibers. All specimens (sleepers) were fabricated following the standard (and daily used) factory production control of MABA Hungária Ltd. The authors executed several laboratory tests (bending tests in the vertical plane) supplemented by DIC measurements, and the experiments were modeled using finite element software to obtain more profound insight. Based on the experimental investigations and numerical modeling, the behavior of the different setups and structures could be compared, and the effects of plastic fiber reinforcement could be evaluated.

Section 2 details the materials and methods, Section 3 contains results and discussion, and Section 4 summarizes the study’s main conclusions.

## 2. Materials and Methods

### 2.1. Materials

#### 2.1.1. Details of the Pre-Stressed Sleeper

**Figure 1:**
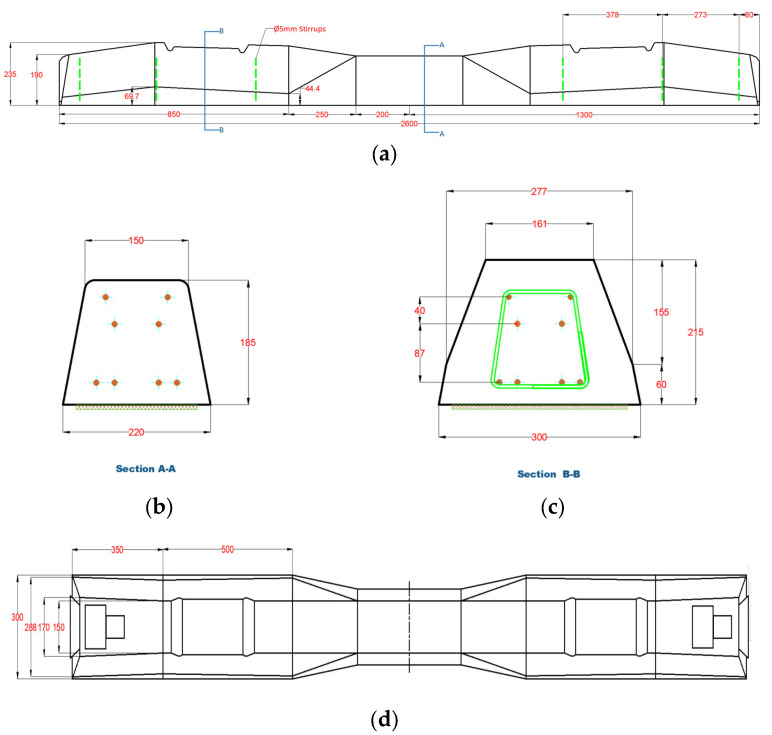
Sleeper details. (**a**) Side view, (**b**) Section A-A, (**c**) Section B-B, (**d**) Top view (all dimensions are given in millimeters).

The employed sleepers were designed with a length of 2600 mm and featured a variable cross-sectional area. The concrete utilized for sleeper construction adhered to the commonly used C50/60 concrete grade specifications, while the reinforcing steel for the sleepers was specified as high-strength steel (as outlined in Section 3). Figure 1 illustrates the sleeper specimen, delineating its geometric dimensions and reinforcement details. Expressly, the sleeper incorporated six ∅5 mm stirrups (B550B) on its sides (∅ means the diameter), and it was longitudinally reinforced with eight ∅6 mm pre-stressed bars (Ap 160/180) to withstand tensile forces.

The concrete sleeper MABA L4 SV 60 railform with a Vossloh System W14 rail fastening system was prepared at the same time as the specimens (for which rail system and with which rail reinforcement system it was prepared is not relevant for the laboratory tests performed), both in normal (regular) and fiber-reinforced design, intended for laboratory tests.

A total of 5056 g of plastic fiber (SIKA HPP50) was used to manufacture the fiber-reinforced concrete sleepers (mixed with 0.8–0.9 m^3^ of concrete per sleeper).

The finished railway sleepers (structural test) and for the cylindrical specimens (hardened concrete test) were delivered to the Building Materials and Structural Testing Laboratory of the Széchenyi István University of Győr after 28 days of hardening.

Regarding tests on hardened concrete specimens, the splitting-tensile strength of cylinder specimens and the compressive strength of cylinder specimens taken during the production of reinforced concrete sleepers for railway construction were tested. These tests were necessary to establish a suitable material model for the subsequent finite-element model and to simulate reality, which can be used to verify the laboratory structural tests. The test results are introduced and detailed in Section 2.1.2 and Section 2.1.3.

#### 2.1.2. Compressive Strength of Cylinder Samples

Compressive strength tests of cylinder samples (determination of the tensile strength of hardened concrete, according to MSZ EN 12390-6 [52]) were carried out. The results are shown in Table 2.

Explanation of the symbols:

RTV reference, compressive strength after storage in water.
RT reference, compressive strength after storage in air.
STV synthetic fiber-reinforced, compressive strength after storage in water.
ST synthetic fiber-reinforced, compressive strength after storage in air.

The results showed that the *f_ci,test_* (compressive strength of cylinder samples) values for reference sleepers were, on average *f_ci,test,reference_* = 78.9 N/mm^2^, while for synthetic fiber-reinforced sleepers *f_ci,test,fiber-reinforced_* = 75.8 N/mm^2^, no significant differences were found in the determination of this value.

Young’s moduli (*E*) for reference (without fiber reinforcement) and fiber-reinforced concrete were determined from graphs constructed from the measurement results and calculated values. The average Young’s modulus for the reference footings was *E_reference_* = 37,825 N/mm^2^, while for the fiber-reinforced cross-sleeper, *E_fiber-reinforced_* = 35,925 N/mm^2^.

#### 2.1.3. Splitting-Tensile Strength of Cylinder Samples

The authors performed the splitting-tensile strength of cylinder samples according to the MSZ EN 12390-3:2019 [53] standard requirements. The results obtained during the studies are shown in Table 3.

Explanation of the symbols:

RBV reference, splitting-tensile strength after storage in water.
RB reference, splitting-tensile strength after storage in air.
SBV synthetic fiber-reinforced, splitting-tensile strength after storage in water
SB synthetic fiber-reinforced, splitting-tensile strength after storage in air.

As before, reference and synthetic fiber-reinforced cylinder samples were prepared for this test. The authors calculated *f_cti,sp,test_* (splitting-tensile strength of cylinders). For reference specimens, the mean splitting-tensile strength was *f_cti,sp,test,reference_* = 5.3 N/mm^2^, while for synthetic fiber-reinforced cylinder samples, the mean value was *f_cti,sp,test, fiber-reinforced_* = 4.6 N/mm^2^.

The results presented in Table 2 and Table 3 served as input parameters for determining the concrete-material parameter of the finite-element model.

### 2.2. Methods

#### 2.2.1. GOM Aramis DIC Measurement System

The GOM Aramis Digital Image Correlation (DIC) system is a sophisticated tool designed for precise measurement and analysis in the field of materials and structural testing. This innovative system utilizes digital image correlation technology to provide accurate, non-contact 3D deformation and strain measurements on various objects and surfaces.

At its core, the GOM Aramis DIC system employs high-resolution cameras to capture a sequence of test object images, typically marked with a random speckle pattern. This pattern allows the software to track and analyze the surface deformation accurately. When the object is subjected to mechanical stress, the cameras continuously record its surface, capturing even the smallest deformations and strains.

The strength of the GOM Aramis system lies in its versatility and precision. It can be used in a wide range of applications, from material testing and component testing to validating numerical models in research and development. The system provides valuable insights into material properties, such as elastic modulus, Poisson’s ratio, and limit of elasticity. It is also instrumental in failure analysis, helping engineers and researchers understand the behavior of materials under stress and identifying potential weak points in a structure.

In conclusion, the GOM Aramis DIC system significantly advances material and structural analysis. Its ability to provide detailed, accurate measurements non-invasively makes it an invaluable tool in research and industrial applications, leading to better product design, quality control, and innovation in material science.

The GOM ARAMIS 5M system is an advanced Digital Image Correlation (DIC) tool known for its versatility and material and structural analysis precision. This unique system can perform detailed measurements without physically contacting the object. Here is a rephrased description of its key aspects.

**Figure 2:**
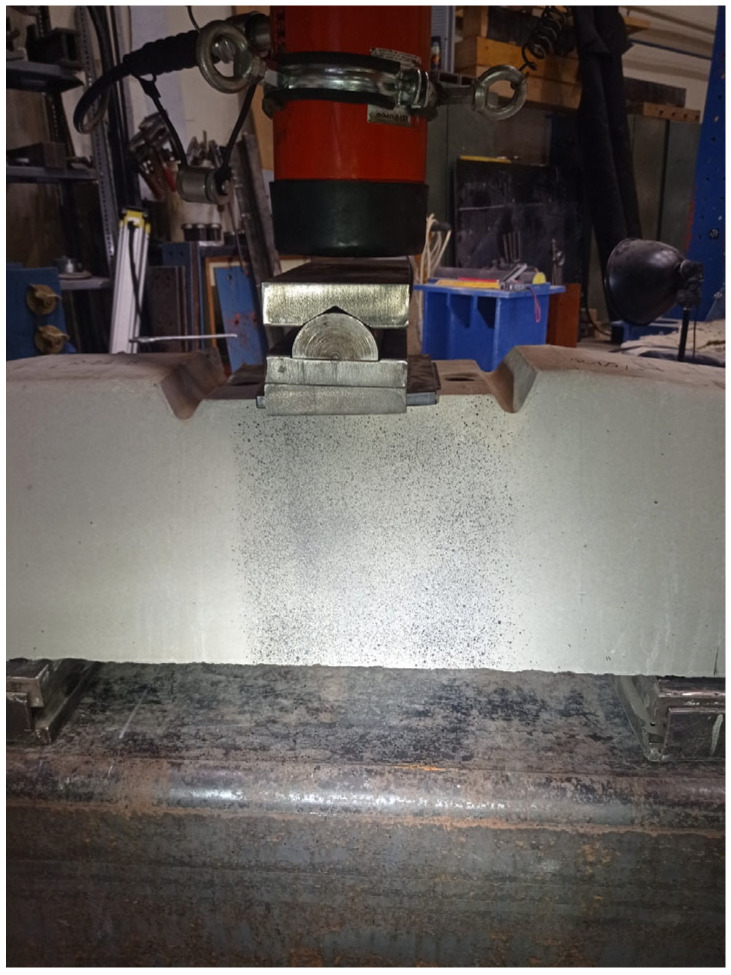
Pattern importance in Digital Image Correlation (DIC) measurements.

ARAMIS systems use DIC technology for non-contact surface and point inspection, which is ideal for delicate or hazardous materials. Suitable for various object sizes, it accurately measures 3D coordinates, movement, and surface changes. It performs detailed analyses of materials’ forming limits and tensile stress responses, which are valuable in material science. Dual 12-megapixel cameras detect minute changes over large areas, providing precise data. Interchangeable camera brackets and calibrated lenses simplify setup for different measurements. The system requires fewer calibrations, ensuring consistent performance. It captures up to 25 fps at full resolution, with adjustable rates for detailed component behavior analysis. ARAMIS uses advanced techniques like normalized cross-correlation and least squares, for accurate nonlinear optimization. It recommends ideal speckle patterns for precise DIC tests and accurate deformation monitoring (see Figure 2). The system continuously monitors gray-level changes and image matching for high-accuracy analyses.

The GOM ARAMIS 5M system stands out for its non-contact, high-resolution, and adaptable measurement capabilities, making it an invaluable tool in various research and industrial applications.

The settings used in this research for the GOM Aramis were as follows. The GOM Aramis 5M DIC system was utilized. The measurement range applied was CP20 or MV90, which can measure an area of 100 × 120 mm from a distance of 836 mm with an accuracy of 0.01 mm. The sampling frequency was 0.5 Hz, approximately 0.5 fps during the measurement. The results were evaluated using GOM Aramis 2018 software.

#### 2.2.2. List of Devices Used for Structural Testing in the Laboratory

Table 4, Table 5, Table 6, Table 7 and Table 8 summarize the devices applied for structural testing in the laboratory.

#### 2.2.3. Static Structural Tests of Sleepers

The measurements were executed according to annexes MÁVSZ 2964:2007 [54] and MÁVSZ 2964/1M [55].

The moment load-bearing requirements of reinforced concrete monoblock sleepers, according to MÁVSZ 2964:2007 [54], at the age of 28 days, are shown in Table 9 for concrete sleeper type L4.

Static tests of sleepers were carried out according to the following list (no dynamic loading test or cyclic loading test was performed):

1 Examination of the cross-section under the rail in the installation position (loading the under-rail cross-section for a positive moment) in accordance with standard MSZ EN 13230-2 [56].
2 Examination of the central cross-section of the sleeper in an inverted position for a negative moment (static loading for a negative moment. Sleeper center (inverted position)) in accordance with standard Section 4.1.3 of MSZ EN 13230-2 [56].
3 Examination of the central cross-section of the sleeper in the normal position for a positive moment (static loading for a positive moment, sleeper center).

Normal and plastic fiber-reinforced sleepers were examined under vertical static loading to observe their behavior under load while recording the progress of the tests using GOM Aramis software (version 2019) and a camera system. The authors continuously monitored and recorded the development of cracks’ images (patterns) during the measurements. During the loadings, the force value was also recorded in minutes for the force ranges and the location, direction, length, and width of the cracks formed. as well as the number and time value of the resulting image in the GOM software (this for later retrieval/verifiability).

#### Examination of the Cross-Section under the Rail in the Installation Position

**Figure 3:**
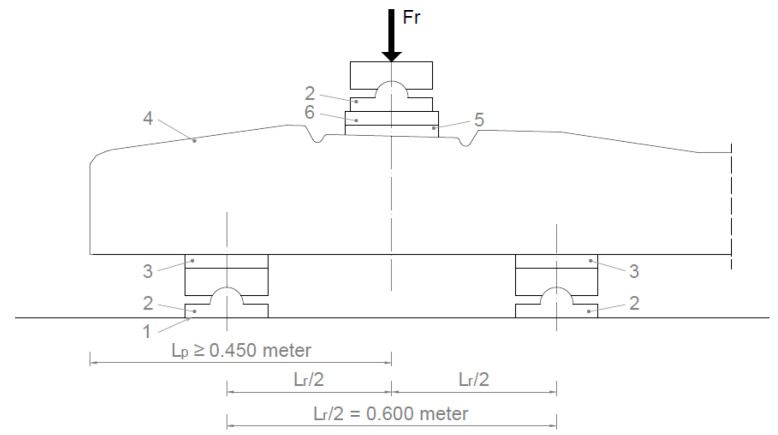
Loading arrangement. Loading of the cross-section under the rail to a positive moment (1. Rigid support; 2. Hinged support. 3. Elastic disk; 4. Pre-stressed concrete sleeper; 5. Standard public insert; 6. Steel sheet with bend; Side stand and washer plate (if required by the customer)).

The load (force) of the under-rail cross-section for a positive moment was performed as follows (see Figure 3).

The initial (reference) forces were calculated according to the formula (Table 9 data).

(1) $$Fr_{o}=\frac{4Mdr}{L_{r}-0.1}\;\left[\text{kN}\right]\;\left(\text{force}\;\text{under}\;\text{the}\;\text{rail}\;\text{for}\;\text{a}\;\text{positive}\;\text{moment}\right)$$

The support distance (*L_r_*) was chosen at 0.6 m.

**Figure 4:**
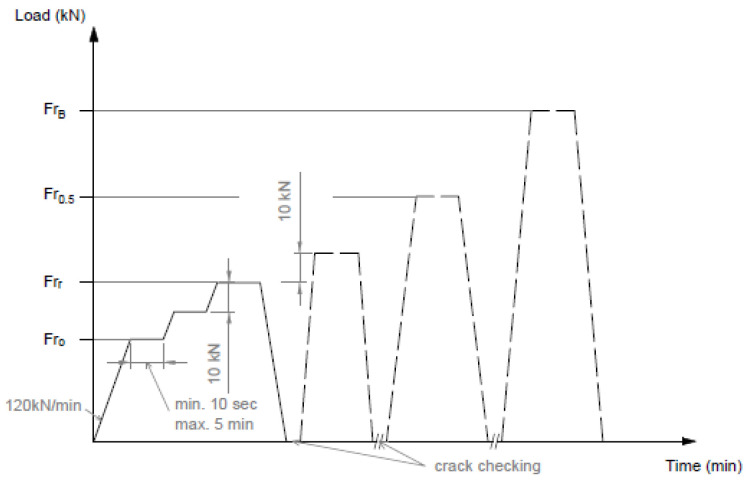
Application of the load. Loading of the cross-section under the rail to a positive moment.

Figure 4 shows the application of the load in a graph.

*F_ro_* value given by the manufacturer based on the required load-bearing capacity;
*F_rr_* the force value that causes the first crack;
*F_r_*_0.10_ the force value causing a crack width of 0.1 mm;
*F_r_*_0.05_ force value causing a crack width of 0.05 mm remaining after unloading;
*F_rB_* a force value that can no longer be increased (causing breakage).

#### Examination of the Central Cross-Section of the Sleeper in an Inverted Position for a Negative Moment

**Figure 5:**
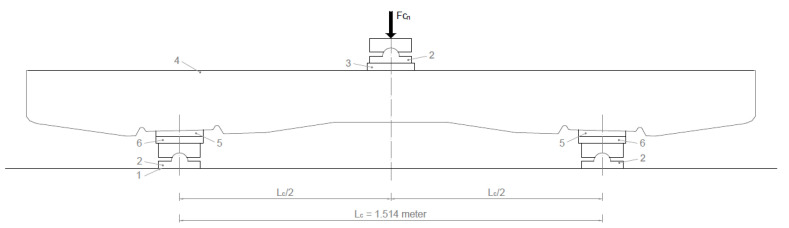
Loading arrangement. Center cross-section loading on a negative moment (1—Rigid support; 2—Hinged support; 3—Elastic disk; 4—Pre-stressed concrete sleeper; 5—Standard public insert; 6—Steel sheet with a bend).

The load (force) for bending tests in the central cross-section of the sleeper in an inverted position for a negative moment was performed as follows (see Figure 5).

**Figure 6:**
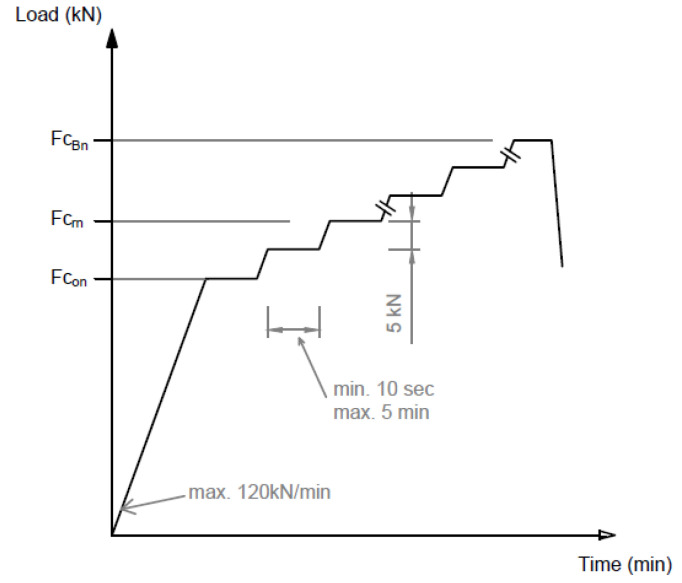
Application of the static load for a negative moment. Sleeper center (inverted position).

Figure 6 shows the application of the load in a graph.

Support distance (*L_c_*): 1.514 m.

(2) $$F_{con}=\frac{4Mdc_{n}}{L_{c}-0.1}\left[\text{kN}\right]\;\left(\text{force}\;\text{at}\;\text{bottom}\;\text{center}\;\text{to}\;\text{negative}\;\text{moment}\right)$$

*F_con_* value given by the manufacturer based on the required load-bearing capacity;
*F_crn_* the force value that causes the first crack;
*F_c_*_0.10__*n*_ the force value causing a crack width of 0.1 mm;
*F_c_*_0.05__*n*_ force value causing a crack width of 0.05 mm remaining after unloading;
*F_cBn_* a force value that can no longer be increased (causing breakage).

#### Examination of the Central Cross-Section of the Sleeper in the Normal Position for a Positive Moment

**Figure 7:**
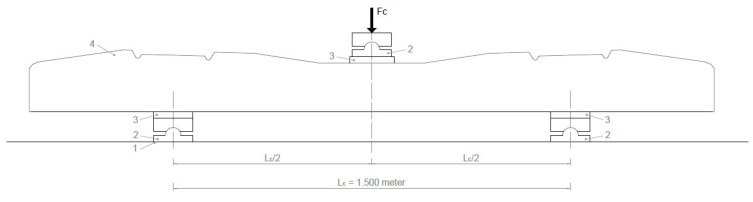
Loading arrangement. Center cross-section loading at a positive moment. (1. Rigid support; 2. Hinged support; 3. Elastic plate; 4. Pre-stressed concrete sleeper).

The load (force) for bending tests in the central cross-section of the sleeper in the normal position for a positive moment was performed as follows (see Figure 7).

**Figure 8:**
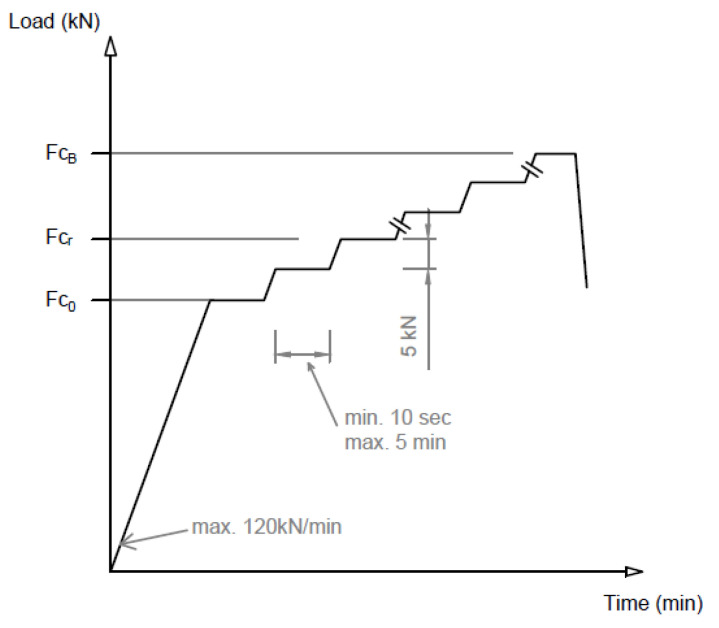
Application of the static load for a positive moment. Sleeper center.

Figure 8 shows the application of the load in a graph.

Support distance (*L_c_*): 1.500 m

(3) $$Fc_{0}=\frac{4Mdc}{L_{c}-0.1}\left[\text{kN}\right]\;\left(\text{static}\;\text{force}\;\text{for}\;\text{a}\;\text{positive}\;\text{moment}\right)$$

*F_c_*_0_ value given by the manufacturer based on the required load-bearing capacity;
*F_cr_* the force value that causes the first crack;
*F_c_*_0.10_ the force value causing a crack width of 0.1 mm;
*F_c_*_0.05_ force value causing a crack width of 0.05 mm remaining after unloading;
*F_cB_* a force value that can no longer be increased (causing breakage).

#### 2.2.4. FE Modeling

##### Concrete Damage Plasticity Constitutive Model (CDM)

In readily available scholarly research and literature, comprehensive expositions of this model are available. At this juncture, a concise overview of the concrete constitutive model is provided, supplemented by relevant details. Utilizing the Prandtl–Reuss concept in conjunction with elasto-plastic deformations, the authors disintegrated the overall strain tensor value $\epsilon_{ij}$ into two distinct components: an elastic component ($\epsilon_{ij}^{el}$) and a plastic component ${(\epsilon }_{ij}^{pl}$), as explicated in the subsequent discussion (see Equation (4)).

(4) $$\epsilon_{ij}=\epsilon_{ij}^{el}+\epsilon_{ij}^{pl}.$$

The internal force–strain relations are precisely defined by the elastic damaged scalar equation (see Equation (5)).

(5) $${\hat{\sigma }}_{ij}=D_{ijkl}^{el}\cdot (\epsilon_{ij}-\epsilon_{ij}^{pl})$$

Subsequently, the symbol $D_{ijkl}^{el}$ represents the diminished elastic stiffness (see Equation (6)).

(6) $$D_{ijkl}^{el}=\left(1-d\right)D_{0}^{el}.$$

In addition, $D_{0}^{el}$ signifies the initial (undamaged) elastic stiffness of the material. Meanwhile, ‘d’ represents the scalar stiffness degradation variable, ranging from zero (indicating an undamaged state of the material) to one (indicating a fully damaged state of the material). In the context of scalar damage theory, the reduction in stiffness is treated as isotropic and is accounted for by the degradation variable ‘d’. By employing the established principles of continuum damage mechanics, the actual internal force is elucidated as follows (see Equation (7)):

(7) $${\bar{\sigma }}_{ij}=D_{0}^{el}\cdot (\epsilon_{ij}-\epsilon_{ij}^{pl})$$

When there is no damage (i.e., *d* = 0), the actual internal force ${\bar{\sigma }}_{ij}$ is equivalent to the internal force ${\hat{\sigma }}_{ij}$. However, as the damage occurs, the actual internal force becomes a more representative measure, particularly when comparing it with the internal force regarding resistance to external loads.

Equation (5) can be reformulated by incorporating the nominal stress and the reduced elastic tensor provided in Equation (7), resulting in Equation (8).

(8) $${\hat{\sigma }}_{ij}=\left(1-d\right)D_{0}^{el}\cdot \left(\epsilon_{ij}-\epsilon_{ij}^{pl}\right).$$

The ensuing internal force–strain relation constitutes the foundation of the damage plasticity constitutive model (see Equation (9)).

(9) $${\hat{\sigma }}_{ij}=\left(1-d\right)\cdot {\bar{\sigma }}_{ij}\to {\hat{\sigma }}_{ij}=\left(1-d_{t}\right){\bar{\sigma }}_{t_{ij}}+(1-d_{c}){\bar{\sigma }}_{C_{ij}}$$

The compression damage variable, denoted as $d_{c}$, and the tension damage variable, denoted as $d_{t}$, evolve from representing the undamaged condition (0) to signifying complete damage (1). Correspondingly, ${\bar{\sigma }}_{t}$ and ${\bar{\sigma }}_{c}$ represent the actual internal force in tension and compression, respectively. Typically, the model used to characterize damage in concrete accounts for two primary failure mechanisms: crushing under compression and cracking under tension.

**Figure 9:**
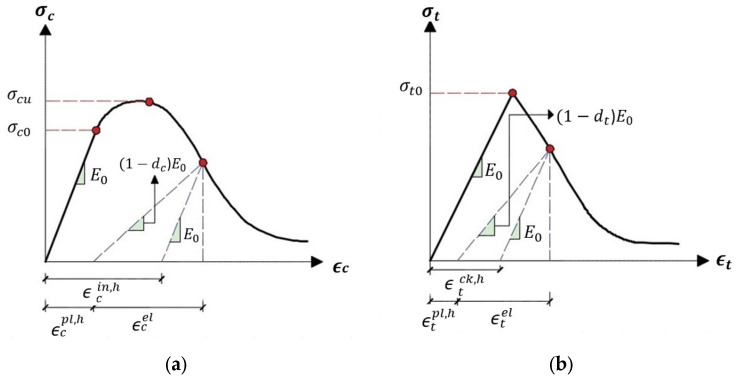
The response of concrete under uniaxial loading conditions in two scenarios: (**a**) compression and (**b**) tension.

However, understanding the uniaxial response of concrete requires a more comprehensive consideration of the intricate degradation mechanisms associated with the cyclic behavior of concrete, involving the opening and closing of micro-cracks. The plasticity-damage model is expected to influence concrete’s uniaxial compressive and tensile responses, as illustrated in Figure 9.

The uniaxial compressive and tensile responses of concrete within the context of the concrete damage plasticity model under both compression and tension loading are described as follows (see Equations (10) and (11)).

(10) $$\sigma_{t}=\left(1-d_{t}\right)E_{0}(\epsilon_{t}-\epsilon_{t}^{pl,h})$$

(11) $$\sigma_{c}=\left(1-d_{c}\right)E_{0}(\epsilon_{c}-\epsilon_{c}^{pl,h})$$

expressing *E*_0_ as the initial (undamaged) Young’s modulus of the material, and denoting $\epsilon_{t}^{pl,h}$ and $\epsilon_{c}^{pl,h}$ as the respective plastic strains in tension and compression, the actual uniaxial compressive and tensile stresses ${\bar{\sigma }}_{t}$ and ${\bar{\sigma }}_{c}$ are presented as follows (see Equations (12) and (13)):

(12) $${\bar{\sigma }}_{t}=\frac{\sigma_{t}}{\left(1-d_{t}\right)}=E_{0}(\epsilon_{t}-\epsilon_{t}^{pl,h})$$

(13) $${\bar{\sigma }}_{c}=\frac{\sigma_{C}}{\left(1-d_{c}\right)}=E_{0}(\epsilon_{c}-\epsilon_{c}^{pl,h})$$

Therefore, the tensile strain $\epsilon_{t}$ is defined as the sum of the plastic strain in tension $\epsilon_{t}^{pl,h}$ and the elastic strain in tension $\epsilon_{t}^{el},$ while the compressive strain $\epsilon_{c}$ is defined as the sum of the plastic strain in compression $\epsilon_{c}^{pl,h}$ and the elastic strain in compression $\epsilon_{c}^{el}$. Accordingly, $\epsilon_{t}^{el}$ and $\epsilon_{c}^{el}$ represent the respective elastic strains in tension and compression.

##### Numerical Modeling of the Sleeper by ABAQUS FE Software

ABAQUS software was utilized in conjunction with the Concrete Damage Plasticity (CDP) model to calibrate the concrete behavior in both the tension and compression states for numerical modeling of the sleeper specimens. Depending on the experimental results of the concrete mixes, CDP was employed to describe the concrete response in tension and compression. Two different mixes were used to cast the experimental sleeper specimens. The first mixture was regular concrete, denoted as “R” in this work, while the other mixture was prepared by adding 5056 g of plastic fibers (SIKA HPP50) and mixing them with 0.8–0.9 m³ of concrete, referred to as “S”. The experimental properties of the two mixes are presented in Table 10, including compression strength (*f_c_*), tensile strength (*f_t_*), and modulus of elasticity *E*.

Moreover, these characteristics were incorporated into ABAQUS to obtain the CDP parameters replicating the desired concrete damage behavior. Following sensitivity analyses, the input CDP parameters were adopted, as presented in Table 11, while the finite element analysis (FEM) assumed a concrete Poisson’s ratio of *v* = 0.2. The impact of the fiber reinforcement within the concrete underwent iterative calibration until the appropriate CDP parameters were established to mirror the experimental behavior.

As previously mentioned, the sleepers were reinforced with pre-stressing tendons. In this study, these tendons had a yield strength of 1650 MPa, a Modulus of Elasticity of 195,000 MPa, and a Poisson’s ratio of *ν* = 0.3. The applied pre-stressed load within the tendons was assumed to be 305 kN.

In contrast, the stirrups had a yield strength of 550 MPa, a modulus of elasticity of 200,000 MPa, and a Poisson’s ratio of *ν* = 0.3. A predefined temperature load was applied to the specified tendons to simulate the pre-stressing effect in ABAQUS.

**Figure 10:**
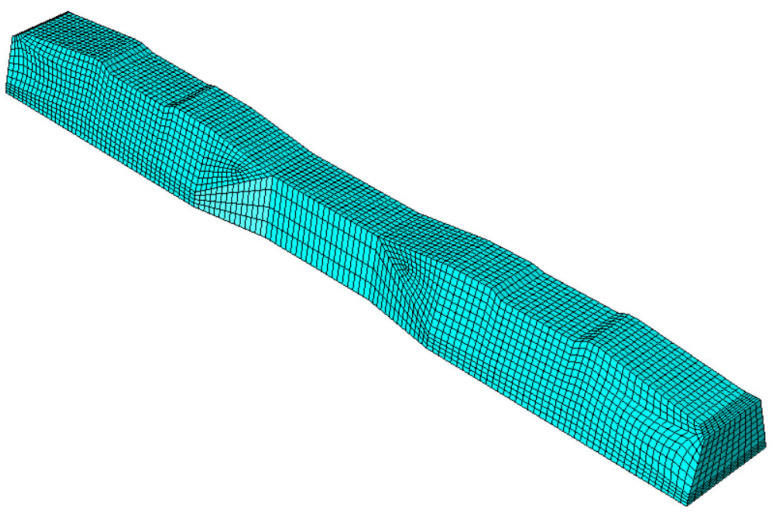
Meshed numerical model in ABAQUS.

Considering that mesh size influences both result accuracy and simulation time, a mesh size study was conducted to assess its impact on accuracy and calculation time. An optimal mesh size of 20 mm was subsequently employed to ensure accuracy. This resulted in a total element count of approximately 13,231 elements, as depicted in Figure 10.

For numerical modeling, an 8-node linear brick element with reduced integration and hourglass control (C3D8R) was utilized to define the concrete material, while two-node 3D truss elements were employed to represent the steel material. The interaction between steel bars and concrete was implemented using the embedded region option.

**Figure 11:**
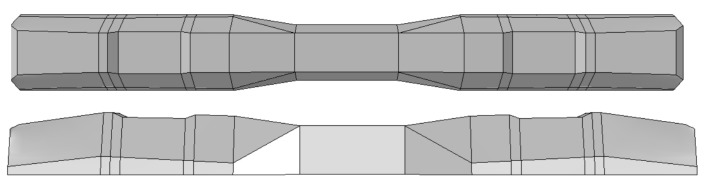

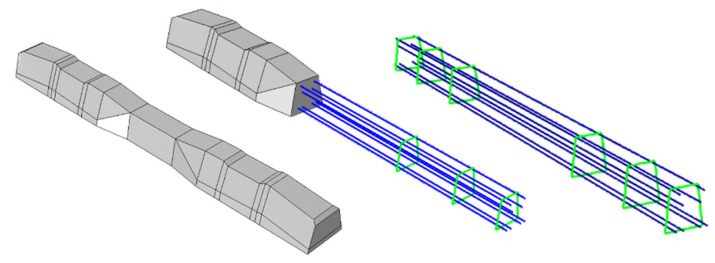
Numerical modeling of the sleeper in ABAQUS.

Furthermore, the numerical analysis involved three steps. The initial step encompassed the establishment of boundary conditions. The second step introduced the pre-stressed (predefined temperature) effect. Finally, the third step focused on analyzing and computing outputs by applying the Static Risk (SR) concentrated force. As a result, the numerical models illustrated in Figure 11 were obtained.

**Figure 12:**
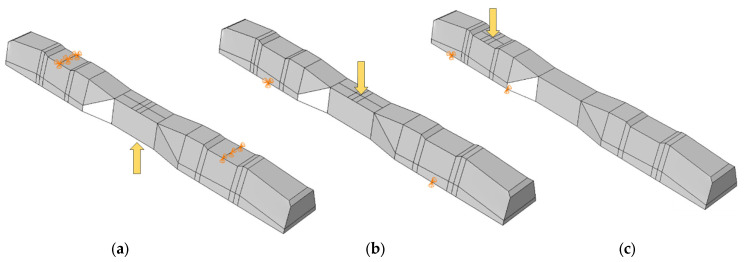
Loading conditions and supporting details related to running in ABAQUS: (**a**) Mid-span upward loading case, (**b**) Mid-span downward loading case, and (**c**) Rail base plate loading case. (The midspan loading case is rotated 180° in reality; this figure is only for comparison and understanding). The arrows mean the concentrated forced applied on the sleepers in the finite element models.

In this research, three distinct test methods were established. In the first method, the sleeper was subjected to a one-point load applied at its mid-span, with the load direction oriented upward. The second method applied a similar one-point load at the mid-span, but the load direction was downward this time. The third case involved loading at the base plate connected to the railway rail. Figure 12 provides a visual representation of all loading scenarios and supporting arrangements.

For the three cases (Figure 12a–c), a concentrated force was applied (static risk) at a specific point. To ensure that the applied force behaves correctly and accurately simulates the experimental conditions, a coupling effect was considered. This coupling effect involves constraining the degrees of freedom (DOFs), and the control point for the coupling aligns with the same point where the loading force is applied.

All loadings and boundary conditions were selected to comply with EN 13230-1 [57] and EN 13230-2 [56] standards, making the sleepers simply hinge supported. In the numerical simulations, the concentrated load was applied vertically to the loading surface at a single point, considering the coupling effect over a defined area. The standard values of *L_r_* (bay length during bending tests) and L_c_ were adhered to, as specified in EN 13230-2 [56], with *L_r_* = 0.6 m and *L_c_* = 1.5 m.

Table 12 presents the experimental results for the tested specimens in each loading scenario. The specimens are denoted as follows:

- R and S specimens correspond to models subjected to rail base plate loading with and without fibers, respectively.
- R_f_ and S_f_ are specimens subject to mid-span upward loading, with and without fibers.
- R_no_ and S_no_ are specimens subjected to mid-span downward loading, with and without fibers.

Table 12 comprehensively presents the ultimate loading values obtained through rigorous experimental testing. These values represent critical insights into sleeper specimens’ structural behavior and load-bearing capacities under various loading conditions and with different concrete mixtures. Alongside this crucial data, the corresponding image is also provided, vividly displaying the areas of damage and cracks that emerged during the experiments. This visual representation serves as a valuable complement to the numerical results, allowing for a more holistic understanding of the sleeper’s response to different loading scenarios and concrete compositions. Using fibers obviously increased the ultimate load slightly, while the cracking severity clearly decreased.

## 3. Results and Discussion

In this section, the authors present in detail the results of the hardened concrete tests of cylindrical specimens, both normal (standard) and synthetic (plastic) fiber reinforced, carried out by sampling during the production of cross beams after 28 days of storage, and the results of the tensile and compressive strength tests of cylindrical specimens.

The extent of crack initiation and the resulting force ranges (stress ranges) were continuously monitored during structural testing. The limit states were recorded on a measurement sheet, and the behavior of the sleepers under load was observed using a GOM Aramis DIC instrument.

### 3.1. Laboratory Tests

#### 3.1.1. Examination of the Cross-Section under the Rail in the Installation Position (Rail Base Plate Loading Case)

**Figure 13:**
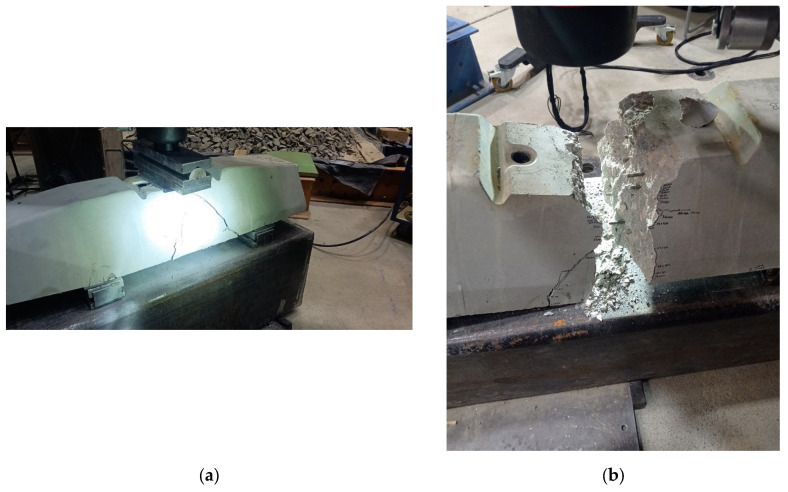
Specimen R1_2. Photos taken during the bending tests related to the examination of the cross-section under the rail in the installation position: (**a**) the test setup with the evolved cracking and (**b**) the broken sleeper due to the loading.

The results of the laboratory experiments considering both types of railway sleepers are presented in Table 12 and Figure 13.

##### Compliance Criteria

Static load, cross-section under the rail:

*F_rr_ > F_ro_*

*F_r_*_0.05_ > *k*_1_*_s_* · *F_ro_*

*F_rB_* > *k*_2_*_s_* · *F_ro_*

#### 3.1.2. Examination of the Central Cross-Section of the Sleeper in an Inverted Position for a Negative Moment (Mid-Span Upward Loading Case)

**Figure 14:**
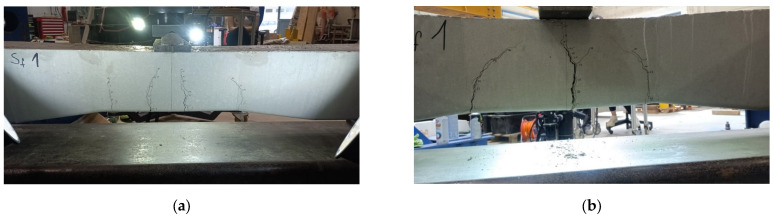
Specimen Sf_1. Photos taken during the bending tests related to the examination of the central cross-section of the sleeper in an inverted position for a negative moment: (**a**) the test setup with the evolved cracking and (**b**) the broken sleeper due to the loading.

The results of the laboratory experiments considering both types of railway sleepers are presented in Table 13 and Figure 14.

##### Compliance Criteria

Static load, middle cross-section:

*F_cm_* > *F_con_*

#### 3.1.3. Examination of the Central Cross-Section of the Sleeper in the Normal Position for a Positive Moment (Mid-Span Downward Loading Case)

**Figure 15:**
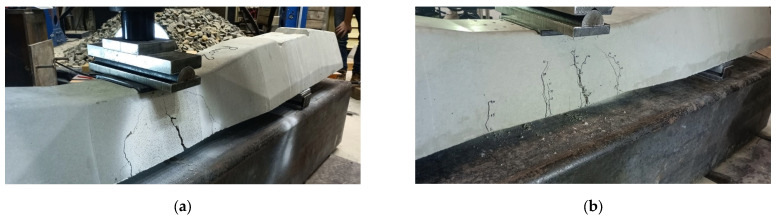
Specimen Rno_2. Photos taken during the bending tests related to the examination of the central cross-section of the sleeper in the normal position for a positive moment: (**a**) the test setup with the evolved cracking from one side and (**b**) the test setup with the evolved cracking from the other side.

The results of the laboratory experiments considering both types of railway sleepers are presented in Table 14 and Figure 15.

##### Compliance Criteria

Static load, middle cross-section:

*F_cm_* > *F_con_*

### 3.2. FE Modeling

#### 3.2.1. Calibration Process

**Figure 16:**
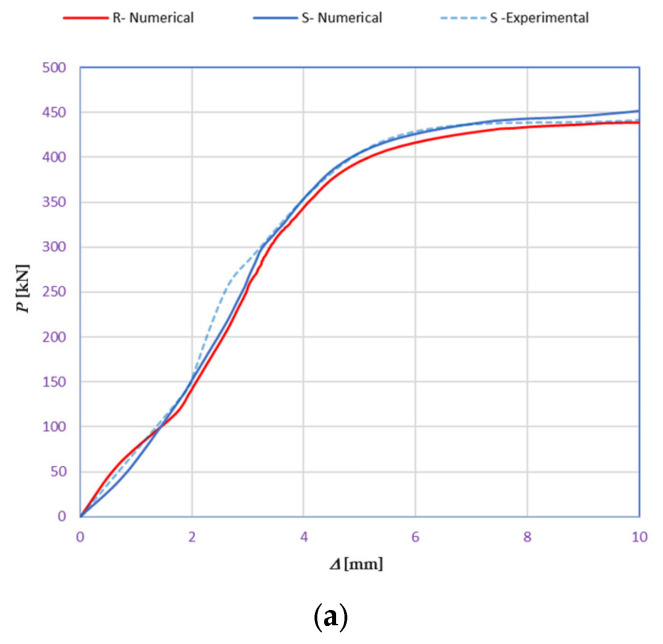

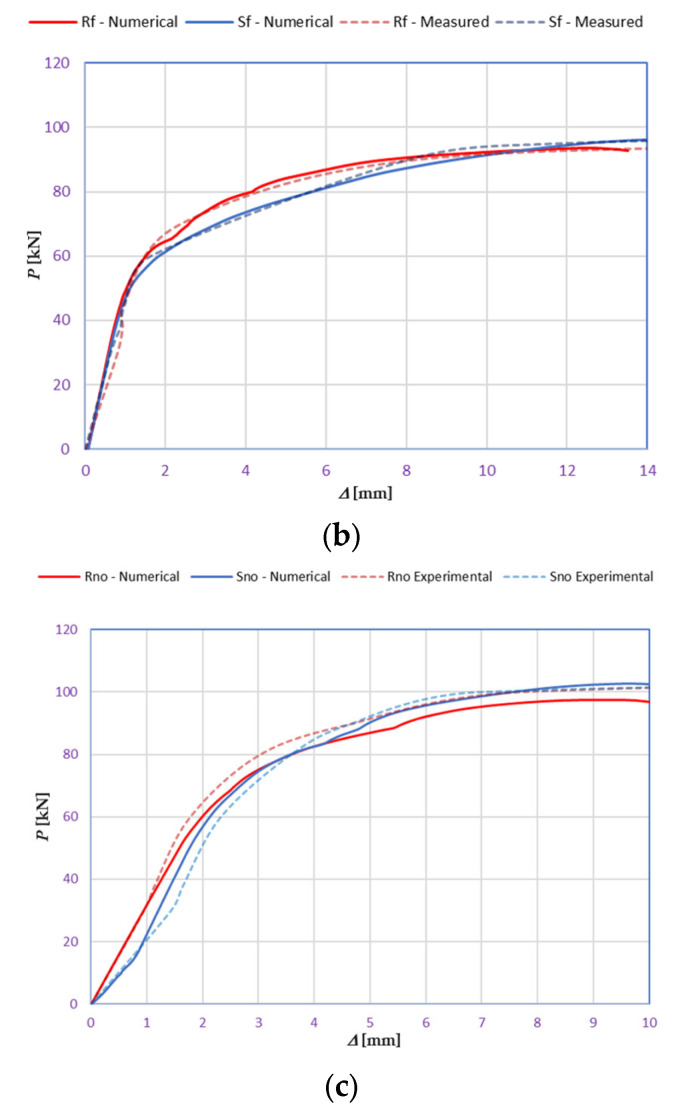
Load-deflection responses of the tested sleepers: (**a**) rail base plate loading case, (**b**) mid-span upward loading case, and (**c**) mid-span downward loading case.

Upon completing the modeling process, the results and plots for calibration and comparison with experimental tests were obtained, as shown in Figure 16. The subfigures depict the load (*P*) versus deflection (Δ) response for all models under various loading conditions and concrete mixtures, where *P* represents the vertical force in kilonewtons (kN) and Δ represents the vertical deflection in millimeters (mm). The calibration was validated by the ultimate load and vertical deflection values from the experiments, with the curves in Figure 16 showing compatibility.

Table 15 details the deflection calibration outcomes, with experimental measurements taken using a specialized device capturing deflection patterns at different loads. The numerical results for both load and deflection align well with the experimental findings. Figure 16 and Table 15 illustrate a detailed comparison between DIC measurements and numerical modeling, confirming the high accuracy of the FEM predictions. For example, in the rail base plate loading scenario, FEM predicted ultimate load and deflection values within 5% of the experimental results. Table 15 further supports this finding by showing that deflection measurements for different load cases, such as mid-span upward and downward loading, were accurately captured by the numerical models. This consistent alignment confirmed that FEM effectively replicated vertical displacement and crack development observed experimentally, validating the combined use of DIC technology and numerical modeling as a robust method for evaluating the structural performance of fiber-reinforced concrete railway sleepers.

#### 3.2.2. Results of FE Modeling

Table 15 represents the calibrated deflection values.

**Figure 17:**
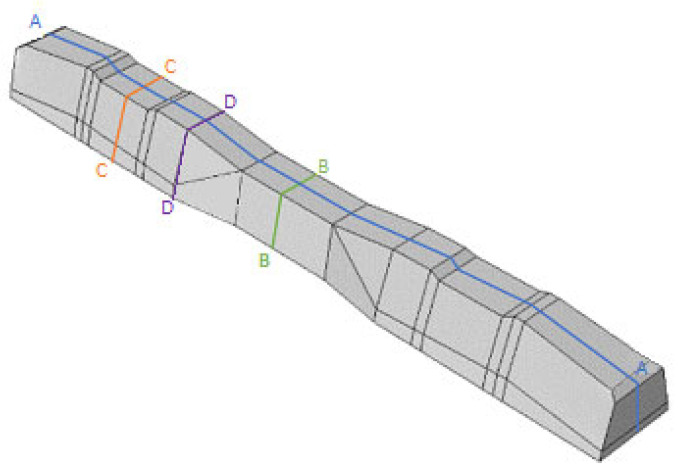
Considered sections of the studied sleepers.

Various sections were examined to facilitate more comprehensive comparisons and provide clarity regarding sleeper behavior, as illustrated in Figure 17. Section A-A divides the sleeper into two-halves along its width. Section B-B crosses the sleeper section at the loading point for mid-span loading scenarios. Section C-C intersects the loading point for side-loaded cases while also crossing the support line for mid-span loading cases. Lastly, section D-D intersects the support line in the case of a side load.

Considering the sections above, the extent of damage and cracking between the numerical and experimental outcomes were compared. Table 16 summarizes the numerical results acquired following the calibration process, with fully dam-aged areas in red and undamaged ones in blue.

Conversely, Table 17 displays stress values for each steel bar in the longitudinal section (A-A) and the loading section (B-B for mid-span loading cases, C-C for side-loading cases). It is worth noting that these cases were derived at the ultimate loading level, resulting in steel bars reaching the yield point or being in a pre-yield stage due to the applied load. Additionally, it was observed that stirrups experienced minimal stresses, as they were positioned beyond the shear range for all loading scenarios.

The curves displayed in Figure 16 provide ultimate load (force) values that align with the experimental findings. Notably, a lower load value was observed when considering mid-span loading compared to the rail base plate loading case. This reduction, which exceeded half of the ultimate loading value, can be attributed to the decrease in the *a*/*d* ratio (the ratio of load to support distance), resulting in higher ultimate load values. The mid-span loading case generally exhibited higher deflection values than the other scenarios. This outcome could be attributed to applying a longer bay length, allowing the sleeper to exhibit a more elastic response, thus reflecting the recorded deflection values. It was evident that the damaged regions primarily occurred between the supports where the highest stresses were initiated. In Table 16, the damaged sections of the models are highlighted in red, signifying the most severely affected areas within the concrete. These color-coded representations specify the intensity of damage, with red indicating fully damaged parts (*d_t_* = 1) and blue hues representing undamaged sections (*d_t_* = 0).

Notably, the most extensive damage was concentrated precisely beneath the applied load, corresponding to the region where the steel bars also experienced heightened stress levels, vividly marked in red, as shown in Table 17. Conversely, the stirrups situated beyond the damage zone exhibited minimal stress, indicated by their unaltered blue color, signifying a case of minimal stress or non-yielding conditions. It is worth noting that the failure type observed is a flexural-shear failure characterized by the generation of inclined cracks or damaged sections in both the experimental and numerical findings. This leads to the conclusion that incorporating stirrups within cracked regions would prove advantageous.

Conversely, an intriguing observation emerges when comparing models with and without fibers. Adding fibers resulted in a marginal increase in the ultimate load values, aligning well with the experimental findings. Furthermore, the corresponding deflection values also exhibited a slight increment in the presence of fibers. However, the most prominent and significant impact in numerical simulations and experiments became evident in the intensity of cracking. Table 16 distinctly reveals that the inclusion of fibers leads to narrower and more confined lines of damage, thereby demonstrating the pivotal role of fibers in constraining and limiting the extent of cracks within the material.

### 3.3. Further Discussion

#### 3.3.1. General Topics

The examination of mechanical properties in pre-tensioned reinforced concrete railway sleepers, both traditional and those enhanced with plastic fiber reinforcement, provides significant insights into their structural performance. Incorporating plastic fibers into sleepers leads to an assumed improvement in crack propagation control and load-bearing capacity. This is evidenced by supposed reduced crack widths and increased ultimate load capabilities in specimens reinforced with fibers, highlighting the fibers’ role in enhancing stress distribution throughout the concrete matrix. Such findings underscore the potential of fiber reinforcement to extend the durability and lifespan of railway sleepers, thereby strengthening the resilience of railway infrastructure.

These results may champion a revolutionary approach to railway sleepers’ design and material selection. Using plastic fibers not only enhances the sleepers’ mechanical properties but also signifies a shift toward greater sustainability within railway infrastructure based on the presumed results. The improvements in crack resistance and load-bearing capacities may indicate that fiber-reinforced sleepers can significantly reduce maintenance costs and increase the service life of railway tracks.

A detailed understanding of stress distribution and deformation patterns under various load conditions has been achieved by accurately calibrating and validating numerical models using ABAQUS finite element (FE) software. These models’ ability to accurately replicate sleeper behavior under a range of stress scenarios highlights the value of advanced simulation techniques in improving the sleeper design and testing process. This advancement not only optimizes the design workflow but also enables the efficient and cost-effective exploration of novel materials and reinforcement strategies.

Incorporating considerations of sustainability into the development of reinforced railway sleepers is an important area of future research. Utilizing plastic fibers, particularly from recycled materials, can significantly contribute to a circular economy and lessen the environmental impact of railway construction. Additionally, lifecycle assessments of fiber-reinforced sleepers can offer valuable insights into their environmental and economic benefits, further promoting sustainable infrastructure development practices.

This research signifies a pivotal progression in civil engineering materials science, offering compelling evidence of the advantages of plastic fiber reinforcement in railway sleepers. It contributes to the evolution of more robust, efficient, and sustainable railway systems globally, overcoming traditional barriers in sleeper design and material application. The ongoing pursuit of innovative materials and design techniques is expected to be crucial in the continual evolution of railway infrastructure.

#### 3.3.2. Specific Topics

Based on the results mentioned above, written in Section 3.1 and Section 3.2, the following observations can be drawn regarding the laboratory tests and calibrated-validated modeling carried out:

- There were cases where fibers were twisted/turned into cracks during/before load removal, but the cracks did not close after load removal;
- The real deflection (vertical displacement) values were compared with the results obtained from numerical modeling;
- In the DIC measurements, the surface area was 100 × 100 mm (or 150 × 150 mm), which showed only a small area of failure. This proved to be a good solution for the cross-section below the rail; however, in the case of loading the middle cross-section up to failure, the cracks appearing in the load “lower pulled-bended area” could not be examined.
- The examination of crack development in pre-stressed concrete railway sleepers reinforced with plastic fibers revealed detailed insights. During load application, cracks first appeared at force values of 45 kN, monitored continuously using DIC technology. The inclusion of plastic fibers significantly reduced crack width and promoted a more confined crack pattern compared to traditional sleepers. For instance, at a force value of 95.2 kN, the plastic fiber-reinforced specimens exhibited less severe cracks, demonstrating their enhanced crack resistance and limited crack propagation. Additionally, the crack width at 0.05 mm remained after unloading at a force of 275 kN for fiber-reinforced sleepers, compared to 320 kN for non-reinforced ones, indicating an improvement in crack control. This crack management significantly contributes to the overall structural integrity and longevity of railway sleepers, highlighting the effectiveness of plastic fiber reinforcement in reducing maintenance needs and extending service life.
- In the case of bending tests of the sleepers with the examination of the cross-section under the rail in the installation position (rail base plate loading case), see Section 3.1.1. The regular (reference) sleepers provided 436.39 kN, while the synthetic-reinforced ones provided 433.83 kN load-bearing capacity considering the average static vertical force values according to the test setup. Comparing the synthetic-reinforced specimens with the regular (reference) ones, there was a –0.6% difference. This means that the plastic reinforcing fibers slightly reduced the reference load-bearing capacity. Interestingly, there was a −6.3% reduction in the *F_rr_* values on average (175.0 kN for R specimens and 164.0 kN for S specimens, respectively), while comparing *F_r_*_0.10_ and *F_r_*_0.05_ values, these differences were +11.6% and −14.1%, respectively. There were no differences between the other results (values) according to the measurements.
- In the case of bending tests of the sleepers with the examination of the central cross-section of the sleeper in an inverted position for a negative moment (mid-span upward loading case), see Section 3.1.2. The regular (reference) sleeper provided 95.2 kN, while the synthetic-reinforced one 97.7 kN load-bearing capacity, considering the average static vertical force values according to the test setup. It was a +2.6% improvement with the synthetic reinforcement. Comparing the *F_c_*_0.05*n*_ values, there was a –14.3% reduction (70.0 kN for the R specimen and 60.0 kN for the S specimen, respectively). There were no differences between the other results (values) according to the measurements.
- In the case of bending tests of the sleepers with the examination of the central cross-section of the sleeper in the normal position for a positive moment (mid-span downward loading case), see Section 3.1.3. The regular (reference) sleeper provided 106.0 kN, while the synthetic-reinforced one had a 111.6 kN load-bearing capacity, considering the average static vertical force values according to the test setup. It was a +5.3% improvement with the synthetic reinforcement. Comparing the *F_c_*_0.05_ values, there was a +33.3% increase (60.0 kN for the R specimen and 80.0 kN for the S specimen, respectively). In the case of Fcr, the improvement was +9.1% (55.0 kN for the R specimen and 60.0 kN for the S specimen). There were no differences between the other results (values) according to the measurements.
- The authors were able to calibrate the FE models (see Section 3.2.1) with a maximum of ±2–5% differences compared to the real experiments conducted in the laboratory (see Section 3.1). The primary calibration process was based on the vertical deflection values measured using the GOM Aramis DIC system.
- Based on the vertical deflection results (see Section 3.2.1), there was a clear result that the specimens reinforced by plastic fibers provided an improvement compared to the regular (reference) types (i.e., without additional plastic fiber reinforcement). Parallel with these results, in the case of bending tests of the sleepers with the examination of the central cross-section of the sleeper in an inverted position for a negative moment (mid-span upward loading case) until 50–55 kN, there was no significant difference, while between this value and approximately 92 kN, the S specimen was worse than the R specimen (the maximal vertical deflection difference was approximately 50%, 6.0 mm compared to 4.0 mm). At approximately 92 kN, there was an “inflection point”, and above 92.0 kN, a slight improvement was observed. In the case of bending tests of the sleepers with the examination of the central cross-section of the sleeper in a normal position for a positive moment (mid-span downward loading case), the inflection point was at approximately 80 kN, i.e., below this value, the S specimen provided lower vertical deflections than the R specimens; hence, above it, the result was the opposite. Below 80 kN, the difference was not significant; however, between 80 kN and the ultimate load, the difference was enormous.
- Section 3.2.2 contains the details related to the FE analysis, and every small detail can be checked synchronously with the DIC measurements.
- Based on the above results, and without long-term field tests, the examined plastic fiber-reinforced sleepers cannot be unequivocally recommended; however, they sometimes improve the load-bearing capacity values and the vertical deflections. More detailed investigations are needed in the future.

## 4. Conclusions

- The current study represents a significant advancement in understanding and using additional plastic fiber reinforcement in manufacturing pre-tensioned reinforced concrete railway sleepers, showing notable progress in railway infrastructure technology.
- Through detailed experimental and numerical analysis, including sophisticated finite element modeling in ABAQUS and comparisons with lab results, this study highlights the structural advantages and resilience that plastic fibers add to concrete sleepers, while also pointing out the complexity and varying impacts of this type of reinforcement.
- The research delves into how these fibers behave under and after stress, uncovering instances where fibers bend or twist into cracks but fail to return to their original shape once the stress is removed. This reveals a complex relationship between the fibers’ assumed orientation and the concrete’s integrity when under pressure.
- The study closely compares actual deflection measurements to those predicted by numerical models using the Digital Image Correlation (DIC) method for accurate vertical displacement measurements. This approach, which focuses on specific surface areas, was effective in identifying failure modes, particularly in crucial sections beneath rail bases, although it did reveal some limitations in tracking crack development under certain stress conditions.
- In terms of quantitative analysis, the research provides an in-depth look at how reinforced sleepers perform under various loads. For example, in bending tests at the rail cross-section, sleepers reinforced with synthetic fibers showed a slight reduction in load-bearing capacity of 0.6% compared to standard sleepers, suggesting a minor decrease in load-bearing ability. However, this was accompanied by exciting findings in reducing and improving specific force resistance values, indicating that while the overall capacity slightly diminished, the performance under certain stresses could actually improve.
- The study also examined different loading scenarios according to related standards, like mid-span upward and downward loading cases, and found improvements in load-bearing capacity with synthetic reinforcement by 2.6% and 5.3%, respectively. These scenarios also showcased variances in vertical deflection performance, with reinforced sleepers displaying both enhancements and decreases in deflection resistance at various stages of loading, highlighting the intricate performance traits of these materials under different structural stresses.
- The accuracy of the Finite Element (FE) models, calibrated to within a 2–5% difference from actual lab experiments, showcases the precision of the simulation techniques used. This calibration, primarily based on vertical deflection measurements taken by the GOM Aramis DIC system, provides a valuable tool for predicting sleeper performance under a range of conditions. However, it also emphasizes the need for more detailed studies and long-term field tests before recommending the widespread use of plastic fiber-reinforced sleepers.
- This thorough study significantly contributes to the field of civil engineering materials science and opens the door to more sustainable and resilient railway infrastructure solutions. By incorporating innovative materials like plastic fibers into the design of railway sleepers, the research underlines a dedication to improving durability and efficiency, establishing a new benchmark for railway construction and maintenance. Although the findings are encouraging, they underscore the essential role of material science and engineering in meeting modern challenges and driving forward technological advances in the railway industry.
- The authors conclude that, based on the presented results and without long-term field tests, the examined plastic fiber-reinforced sleepers cannot be unequivocally recommended. However, they sometimes improve load-bearing capacity values and vertical deflections. More detailed investigations are needed in the future.

## Figures and Tables

**Table 1:** The details of the executed literature review, with the consideration of relevant aspects.

Ref.	Type of the Tested/ Investigated Railway Sleepers	Material of the Sleepers	Tensioning	Additional Fiber Reinforcement	Type of Additional Fibers	Quantity of the Fibers	Investigation Type	Aims and Methods	Comparisons	Main Conclusions
[21]	Carbon Fiber Reinforced Polymer Concrete (CFRPC) sleeper and Polymer Concrete (PC) sleeper	Recycled chopped carbon fiber and epoxy resin	Not applicable	Yes	Carbon fibers	2 wt% carbon fiber for optimal results	Laboratory experiments including flexural strength tests and impact vibration tests. Three-point bending test and impact test for measuring stiffness and damping properties	The aim was to reduce vibration and noise problems in railway slabs using CFRPC. Additional methods: oxygen plasma treatment to enhance the adhesion between carbon fiber and epoxy resin, X-ray photoelectron spectroscopy (XPS) analysis, and scanning electron microscopy (SEM). Validation phases: the study included validation through comparison with polymer concrete sleepers using a 1/6 scale model of the railway structure	Comparisons were made between CFRPC sleepers and polymer	The CFRPC sleeper with 12 mm fiber length and 2 wt% carbon fiber content exhibited the highest mechanical and dynamic properties, showing a flexural strength of 25.12 MPa and a damping value of 0.01954. This configuration reduced the noise level by 4 dB compared to the PC sleeper
[22]	B70, pre-stressed concrete sleeper and a new CFRP (Carbon fiber-reinforced polymer)-reinforced concrete sleeper	Pre-stressed concrete sleeper: steel-reinforced concrete; CFRP-reinforced concrete sleeper: carbon fiber-reinforced polymer (CFRP) laminate reinforcements	B70 sleeper: pre-stressed (pre-tensioned); CFRP-reinforced sleeper: without pre-stressing	B70 sleeper: no; CFRP-reinforced sleeper: yes	B70 sleeper: not applicable. CFRP-reinforced sleeper: carbon fiber	N/A	Laboratory experiments: rail seat static positive moment tests, modal and harmonic response analysis. Numerical simulation: finite element simulation using ANSYS^®^ 2020 R1 software	The aim was to compare the modal and harmonic response of new CFRP laminate-reinforced concrete sleepers with B70-type pre-stressed sleepers. The study aims to evaluate damping capabilities and the potential for reducing railway maintenance costs and extending service life. Other methods: validation phases with laboratory experiments, comparison with results from the literature	Comparisons were made to B70-type pre-stressed sleepers which are widely used worldwide for high-speed train lines	The CFRP-reinforced non-pre-stressed sleepers demonstrated higher damping capabilities compared to the pre-stressed B70 sleepers. The non-pre-stressed sleepers showed a significant reduction in stress at critical frequencies, resulting in a potentially longer service life and reduced maintenance needs. Specifically, stress levels in non-pre-stressed CFRP sleepers were kept below 16 MPa in critical frequency ranges, whereas pre-stressed B70 sleepers experienced stress levels up to 212 MPa, which would likely reduce their service life
[23]	Unknown-type concrete sleeper	Synthetic fiber-reinforced concrete	N/A	Yes	A hybrid combination of steel and polypropylene fibers. Synthetic fibers of different geometry and form	Different quantities were tested: 2.0 kg/m^3^ and 3.0 kg/m^3^	Laboratory experiments including three-point bending tests (3PBT) to assess flexural tensile strength	The aim was to assess the flexural tensile strength of concrete with synthetic fibers and to propose a new formula for flexural tensile strength calculation. The study also evaluated the ductility and residual flexural tensile strengths of fiber-reinforced concrete	Comparisons were made with plain concrete and with other studies on fiber-reinforced concretes	The addition of synthetic fibers to concrete increased the flexural tensile strength by 5.5% to 13.5% depending on the mixture. The best results were obtained with a hybrid blend of fibers. The ductility and residual flexural tensile strengths were also significantly enhanced with the incorporation of synthetic fibers
[24]	Unknown-type concrete sleeper	The sleepers tested are Laminated form carbon fiber-reinforced polyurethane-reinforced concrete sleepers	According to the provided information, it is suggested that pre-stressing might not be necessary for the L-CFRPU-reinforced concrete sleepers	Considering the basis/normal tendons, the sleepers contain additional fiber reinforcement	The additional fiber reinforcement type (material) is carbon fiber-reinforced polymer, specifically Laminated form carbon fiber-reinforced polyurethanes	N/A	The type of investigation done includes incremental LUR tests, which are laboratory experiments to test the mechanical properties of the sleepers	The aim of the investigations was to test the new design of L-CFRPU-reinforced concrete sleepers and compare them with widely used concrete sleepers in terms of design load capacities and service life; no specific mention of a validation phase is provided in the excerpt	The study involved comparisons of newly produced sleepers with the traditional concrete sleepers, potentially with an emphasis on load capacities and longevity	The main conclusions of the paper regarding the additional fiber-reinforcement are: newly produced L-CFRPU-reinforced concrete sleepers show higher enough design load capacities without needing pre-stressing, suggesting a longer service life compared to traditional concrete sleepers
[25]	The paper considers two types of sleepers: standard B70 pre-stressed concrete sleepers and the new LCR-6-type sleepers	The B70 sleepers are made of steel-reinforced concrete, whereas the LCR-6 sleepers consist of laminated carbon fiber-reinforced polyurethane materials without a pre-stressing process	B70 sleepers are pre-stressed concrete sleepers, while LCR-6 sleepers are non-pre-stressed concrete sleepers reinforced with carbon fiber	LCR-6 sleepers contain additional fiber reinforcement, while B70 do not	The additional fiber reinforcement material in LCR-6 sleepers is carbon fiber	N/A	The investigation type was laboratory experiments that included modal analysis, rail seat loading tests, and repeated impact tests	The aim of the investigation was to evaluate the impact damping characteristics and mechanical strength of LCR-6 sleepers compared to B70 sleepers, also employing modal analysis. Validation phases, such as feasibility studies and mechanical tests, were applied alongside impact loading procedures	Comparisons were made between the conventional B70 pre-stressed concrete sleepers and the new LCR-6-type sleepers after they were subjected to repeated impact loads	In the paper, it is concluded that the LCR-6 sleepers demonstrate an 83% reduction in FRF magnitude values compared to B70 sleepers after impact loads and show an increase of damping ratios by 274%. The cracks formed on impacted LCR-6 sleepers were minor and did not cause significant mechanical capacity loss
[26]	Unknown-type concrete sleepers	The sleepers discussed are concrete sleepers, specifically laminated carbon fiber-reinforced polyurethane-reinforced concrete railway sleepers, as opposed to standard steel-reinforced concrete sleepers	The newly developed sleepers are mentioned as an alternative to pre-stressed (pre-tensioned) concrete sleepers but do not explicitly state whether the new type is pre-stressed or not	Yes, the newly developed sleepers contain them	Carbon fiber-reinforced polymer	N/A	The study conducted laboratory experiments, including improved incremental loading—unloading-reloading tests and one-stage static loading tests	The aim of the investigations was to assess the performance of new laminated carbon fiber-reinforced polyurethane-reinforced concrete railway sleepers under demanding operational conditions. The tests were used to measure sleeper deformations, first crack formation loads, and permanent crack width, but no explicit mention of a validation phase is made	The new LCR sleepers were compared to ordinary pre-stressed concrete sleepers	The study concluded that the new LCR sleepers met the static capacity requirements of standards and exhibited fewer vertical plastic deformations, which are crucial for high-speed railways. Sleepers that had the L-CFRPU surface sanded and a more spread of L-CFRPU in the concrete section showed better performance than the other samples
[27]	Unknown concrete sleeper	Concrete and polymer composites	Pre-stressed concrete sleepers	The paper discusses polymers reinforced with glass fibers as an alternative material	Glass fibers	N/A	Laboratory experiments and evaluation of thermal expansion properties	The aim was to assess the suitability of polymers and polymer composites for sleeper production, especially in terms of thermal expansion and gauge stability. Experimental measurement of thermal expansion in laboratory conditions was mentioned	Comparison between traditional materials and the tested polymer composite	The use of glass fibers as reinforcement in polymers reduced the thermal expansion coefficient, indicating a positive impact on the stability of the sleeper’s dimensions under temperature changes
[28]	Unknown-type concrete sleeper	Steel-reinforced concrete	N/A	The concrete contains additional fiber reinforcement	Glass fiber	The paper mentions various replacement ratios of sand aggregates with GFRP waste fractions up to 15% in weight of total aggregates	Investigation type: laboratory experiments. Tests executed: compressive and flexural strength tests were executed	The aim was to assess the reuse of GFRP wastes in concrete-based materials, focusing on their mechanical properties and cost-effectiveness. Research methods: mix design formulations, compressive strength tests, flexural strength tests, and the incorporation of silane coupling agents were applied. Validation phases: the paper does not explicitly mention validation phases but includes extensive testing and comparison of results	Comparisons were made with concrete mixtures containing varying amounts of GFRP recyclates and other fillers	Incorporation of GFRP recyclates in concrete materials improves compressive and flexural behavior up to 15% replacement ratios. However, higher replacement ratios may lead to significant losses in mechanical properties due to the increased water-cement ratios needed for workability
[29]	Unknown-type concrete sleeper	The material of the considered sleeper is fiber-reinforced concrete, specifically with Fiber-Reinforced Polymer	The reinforcement type for the concrete sleeper in this case is not clearly stated in the provided excerpts, so it remains unspecified. However, the context of high-speed railway applications suggests that they are likely to be pre-stressed for added strength and durability	The sleepers contain additional fiber reinforcement in the form of Fiber-Reinforced Polymer-Optical Fiber	The sleepers contain additional fiber reinforcement in the form of Fiber-Reinforced Polymer-Optical Fiber	N/A	The investigations done include both laboratory experiments and field dynamic tests to monitor sleeper dynamic strain. There was also the use of a finite element model to simulate sleeper strain under loading conditions	The aim of the investigations was to monitor the health of the track structure, specifically the sleeper dynamic strain, as a means to reflect the wheel/rail force under high-speed train loads. To achieve this, a Fiber-Reinforced Polymer-Optical Fiber sensor was embedded within the sleeper. The laboratory tests and field tests served as validation phases for the monitoring method	Comparisons to other structures are not directly mentioned in the excerpts provided. The study primarily focuses on validating the embedded FRP-OF sensor within the sleeper itself	The main conclusion regarding additional fiber reinforcement is that the embedded FRP-OF sensors show good performance for monitoring dynamic strains of bi-block sleepers, offering a valuable supplement to future wheel/rail force monitoring. The sensors enabled the detection of strain variations that were in line with the characteristics of wheel load distribution ratio
[30]	CC16: Conventional pre-stressed concrete sleeper used in the Korean railway system. BS16: Same geometry and reinforcement as CC16, but with partial replacement of Portland cement with GGBFS. BSF16: Same as BS16 but with 0.75% steel fibers and removal of stirrups. BS14: Same as BS16 but with 14 pre-stressing strands. BSF14: Same as BSF16 but with 14 pre-stressing strands	Steel-reinforced concrete	pre-stressed	CC16: No. BS16: No. BSF16: Yes. BS14: No. BSF14: Yes	Hooked steel fibers	BSF16 and BSF14 contain 0.75 vol% hooked steel fibers	Laboratory experiments were conducted including static and impact tests using a drop-weight impact test machine with potential energies of 7.85 and 9.81 kJ. Tests included measuring maximum load, deflections, strain variations, and residual flexural performance	The aim was to investigate the effects of GGBFS and steel fibers on the flexural behavior of railway PSC sleepers under static and impact loads, and to evaluate the structural integrity after impact damage. Research methods: static bending tests, drop-weight impact tests, measurement of deflections, strain, and residual flexural performance	Comparisons were made between sleepers with different materials and reinforcement configurations. For example, CC16 was compared to BS16 and BSF16 under static and impact loads	The addition of 0.75 vol% steel fibers improved the impact resistance and residual capacity of the PSC sleepers. Specifically, BSF16 showed 22% and 87% higher residual flexural strengths at potential energies of 7.85 and 9.81 kJ, respectively, compared to BS16 without fibers
[31]	Unknown-type ultra-high performance concrete sleeper	Ultra-high performance concrete with steel fiber reinforcement	The UHPC sleepers were fabricated using the post-tensioning method	The sleepers contain additional steel fiber reinforcement	Steel fibers are used as the supplementary fibers	Steel fibers are used as the supplementary fibers	Steel fibers are used as the supplementary fibers	The research aimed to investigate the structural and electrical responses of UHPC sleepers with different steel fiber contents. The tests conducted provided an evaluation of the mechanical properties and electrical insulation performance of the sleepers	The paper compares the performance of UHPC sleepers with different levels of steel fiber content but does not explicitly compare them to sleepers made of other materials or with no steel fiber reinforcement	The study concluded that UHPC sleepers with steel fiber content greater than 1% showed improved control over early-stage crack development. Additionally, the research highlighted a strong correlation between the volume fraction of steel fibers and the structural behavior of the sleepers, emphasizing the benefits in tensile capacity and fatigue performance
[32]	Unknown-type concrete sleeper	Macro Synthetic Fiber-Reinforced Concrete (MSFRC)	Pre-stressed	Yes	Macro synthetic fibers	N/A	Laboratory experiments with full-scale dynamic impact behavior studies, and a series of material-scale flexural experiments	The aim was to evaluate the residual performance of MSFRC sleepers after dynamic impacts from wheel-rail irregularities. Research methods included full-scale testing and analysis of residual flexural strength and toughness. Validations were made through comparisons with conventional sleepers	Comparisons made with conventional pre-stressed concrete sleepers	The addition of macro synthetic fibers increased the residual flexural strength and toughness of MSFRC sleepers, making them more adaptable to the rail network with improved residual performance after dynamic impacts when compared to the conventional pre-stressed concrete sleepers
[33]	Pre-stressed Geopolymer Railway Sleepers (PGRS) and Steel Fiber-Reinforced Geopolymer Railway Sleepers (SFRGRS)	Geopolymer concrete composed of Fly Ash (FA) and Ground Granulated Blast Furnace Slag (GGBFS), with steel fibers used as secondary reinforcement	Pre-stressed	Yes	Steel fibers	1% by volume	Laboratory experiments: static bending tests, rail seat bending moment evaluation, compressive strength tests, flexural strength tests, modulus of rupture tests, electrical impedance tests, and microstructural analysis using SEM-EDAX & XRD	The aim was (i) to assess the load-carrying capacity, ductility, and durability of the proposed geopolymer concrete sleepers; (ii) to Evaluate the impact of steel fiber reinforcement on the mechanical properties and structural performance of the sleepers; (iii) to compare the performance with conventional pre-stressed concrete sleepers (PCS). Validation Phases: experimental validations were performed on full-scale sleeper members under static loading conditions	Comparisons were made between: (i) steel fiber-reinforced geopolymer sleepers (SFRGRS), (ii) pre-stressed geopolymer sleepers (PGRS) and (iii) conventional pre-stressed concrete sleepers (PCS)	The addition of steel fibers to geopolymer concrete significantly enhances its mechanical properties. The SFRGRS sleepers showed a 23% increase in load-carrying capacity and a 24.21% increase in moment capacity compared to conventional concrete sleepers (CC). The inclusion of steel fibers improved the displacement ductility demand by 25% (ultimate), 28.5% (failure), and 3% (residual ductility index). This indicates a significant enhancement in the ductility and durability of the sleeper members
[34]	Unknown-type concrete sleeper	Steel-reinforced concrete	Pre-stressed	Yes	Steel fibers	Hybrid steel fibers of 0, 0.3, 0.5, 0.7, and 1% by volume	Laboratory experiments including static tests like negative bending moment tests	The aim was to investigate the efficiency of using steel fibers in improving sleeper characteristics such as load-carrying capacity and energy absorption, with a focus on service life improvements for high-speed tracks. Validation was done by averaging results from two specimens for each condition	Comparisons to conventional sleepers without additional steel fibers were made	The main conclusion is that the use of hybrid steel fibers in concrete sleepers leads to an increase in load-carrying capacity and energy absorption. This results in enhancements in the service life of the sleepers, while maintaining dynamic characteristics similar to conventional sleepers
[35]	Type: BS16, BS14, BSF16, BSF14, CC16 (Conventional railway sleepers)	Ground granulated blast furnace slag (GGBFS) and steel fiber-reinforced concrete	Pre-stressed	Yes	Steel fibers	0.75% by volume	Laboratory experiments and field tests. Static bending tests, fatigue tests, pull-out tests for the rail fastening shoulder	Aim: To investigate the influence of steel fibers and GGBFS on the static and fatigue performance of pre-stressed concrete sleepers, and to assess the potential to reduce the number of pre-stressing strands. Methods: Static and fatigue testing at the rail seat section and center section, compared with conventional sleepers. Validation: Yes, the results were compared with existing standards and criteria	Comparisons were made with conventional sleepers without steel fibers (CC16)	Sleepers incorporating 0.75% steel fibers showed higher loads at first cracking and improved crack control, leading to enhanced flexural and fatigue performance. Steel fibers effectively replaced traditional stirrups and provided an economical solution by allowing the use of fewer pre-stressing strands
[36]	Non pre-stressed Ultra-high performance fiber-reinforced concrete (UHP-FRC) sleeper	Ultra-high performance fiber-reinforced concrete (UHP-FRC), also known as reactive powder concrete (RPC)	Without pre-stressing tendons	Yes	Macro steel fibers	Optimal content of macro steel fibers: 2% by volume	Laboratory experiments. Tests: 7-day compressive strength, 28-day flexural strength, modulus of elasticity, flexural (modulus of rupture) tests, compression tests on cylinder samples, elastic modulus determination	The aim was o develop an optimal UHP-FRC material with high flexural strength for manufacturing non-pre-stressed concrete sleepers. The applied methods were the Taguchi method for optimization, trial mixes, and material testing procedures. The validation was related to material tests to determine the optimal content of fibers and mechanical properties	Comparison with conventional pre-stressed concrete sleepers (PSC) for similar axle loads (25 TAL/ton axle load/and 40 TAL). Found that non-pre-stressed UHP-FRC sleepers have slightly larger cross-sections but satisfy the required shear strength and are more efficient in design and manufacturing	The study concluded that UHP-FRC with 2% of macro steel fibers by volume significantly enhances the compressive and flexural strengths of the sleeper material, achieving 7-day compressive strength of 103 MPa and 28-day flexural strength of 21.4 MPa. The non-pre-stressed UHP-FRC sleepers designed for 25 TAL and 40 TAL are only slightly larger than conventional PSC sleepers and do not require pre-stressing tendons, simplifying the manufacturing process
[37]	Unknown-type concrete sleeper	The study focuses on concrete sleepers, with potential enhancements using glass powder and steel fiber as silica fume replacements	N/A	Yes	Glass powder and steel fiber	Investigated proportions are 0.5%, 1%, and 1.5% steel fiber by concrete volume and 5%, 10%, and 15% glass powder by weight of cement content	Laboratory experiments were conducted, with particular tests not specified in the provided snippets	The aim was to investigate the potential of replacing silica fume with a combination of glass powder and steel fiber in concrete sleepers, and to evaluate the mechanical performance of these alternative admixtures	The study compared the performance of traditional silica fume concrete admixtures with new admixtures containing glass powder and steel fiber	The study concluded that concrete admixture with the coupling of glass powder and steel fiber improves all characteristics of the concrete compared to traditional silica fume admixtures. An optimal combination for mechanical performance was found to be 10% glass powder and 1.5% steel fiber by respective measures
[38]	Unknown-type concrete sleeper	Steel-reinforced concrete	Pre-stressed	Yes	Steel fibers	The volume percentage of steel fibers added is 0.75 vol% as stated in one of the papers	The studies involved laboratory experiments including drop-weight impact test machine and other static and dynamic tests	The aim was to investigate the effects of supplementary materials and fiber reinforcement on the performance of concrete sleepers, such as their durability, impact resistance, and structural behavior. Validation phases are not explicitly mentioned in the provided excerpts	Comparisons made to other sleepers, including those made with conventional materials and varying amounts of steel fibers or different numbers of pre-stressing strands	Adding 0.75 vol% steel fibers and increasing the number of strands significantly enhanced the resistance of pre-stressed concrete sleepers to multiple impacts, improving peak reaction load, deflection behavior, crack width reduction, and concrete spalling prevention. These modifications also led to improved flexural strength and overall performance of the sleepers under impact loads
[39]	Unknown-type concrete sleeper	Steel-reinforced concrete	Pre-stressed	Yes	Polypropylene fiber (PPF)	Quantities of polypropylene fiber used: 0.5 kg/m^3^, 0.7 kg/m^3^, 0.9 kg/m^3^, 1.5 kg/m^3^, 2 kg/m^3^, and 4 kg/m^3^	Laboratory experiments and tests were conducted. Types of tests included compressive strength, splitting tensile strength, three-point flexural strength, Vee-Bee consistometer (VB) tests, Rapid Chloride Penetration Test (RCPT), water penetration, ultrasonic tests, and sorptivity tests	The aim of the investigations was to assess the effect of polypropylene fiber on the durability, and physical and mechanical characteristics of concrete for application in sleepers. Additional methods included Scanning Electron Microscope (SEM) analysis and X-ray Diffraction (XRD) to study microstructures and interfacial transition zones. Validation phases included the comparison of results with known standards and control samples	Comparisons were made with plain concrete sleepers (PC1) and various concentrations of polypropylene fiber-reinforced concrete (PPFRC) sleepers	The addition of polypropylene fiber reduced compressive strength but improved splitting tensile and flexural strengths, with the optimum amount being 0.7 kg/m^3^. At this concentration, compressive strength was 8.8% lower, tensile strength was 39% higher, and flexural strength was 10% higher compared to plain concrete sleepers. Polypropylene fibers also significantly improved the durability of the concrete by reducing chloride penetration, water penetration, and sorptivity attributed to the pore-blocking effect of the fibers
[40]	Unknown-type concrete sleeper	Macro synthetic fiber-reinforced concrete	N/A	Yes	Macro synthetic fibers	N/A	The research included laboratory experiments to study the static and dynamic behavior of MSFRC under loading for sleeper applications	The aim of the investigations was to evaluate the performance of MSFRC under static and dynamic loadings and to assess its economic feasibility for railway sleeper applications through life cycle costing; other relevant research methods such as life cycle cost analysis were applied. Validation process was not specified	Comparisons were made to conventional sleeper materials in the life cycle cost analysis to identify the most financially viable option for railway sleepers	Fibers have little impact on static compressive and pre-cracking flexural strengths but considerably improve post-cracking behavior, energy absorption, and ductility. At higher fiber dosages, workability, and compressive strength may decrease due to the balling effect
[41]	Pre-stressed concrete sleeper with ground granulated blast furnace slag (GGBFS) and steel fibers	Steel-reinforced concrete with GGBFS and steel fibers	Pre-stressed	Yes	Steel fibers	0.75% by volume of steel fibers	Laboratory experiments and field tests: static flexural tests (third-point bending); impact loading tests (drop weight impact tests); chloride migration tests; accelerated carbonation tests; freeze-thaw resistance tests	The aim was to evaluate the mechanical properties and durability performance of concrete mixes with GGBFS and steel fibers for railway sleepers, and to assess their eco-friendliness by reducing CO_2_ emissions. Research methods: mechanical property tests, durability performance evaluations, life cycle assessment for CO_2_ emissions, and comparison with conventional concrete mixes. Validation phases: pilot production and mechanical properties evaluation of ninety pre-stressed concrete sleepers under factory conditions were done to assure quality control and validate laboratory findings	Comparisons were made to conventional concrete sleepers (control mix CC) currently used for railway sleepers	The addition of 0.75% steel fibers to slag concrete (GGBFS) resulted in enhanced flexural strength, toughness, and freeze-thaw resistance compared to the mix without fibers. The use of steel fibers decreased the carbonation coefficient by 18% and showed improved durability performance with reduced chloride ion penetration
[42]	Unknown-type concrete sleeper	Steel-reinforced concrete	Pre-stressed	Yes	Carbon fibers (CF), Carbon nanotubes (CNT), Carbon nanofibers (CNF)	N/A	Laboratory experiments and numerical simulation. Three-point bending tests and static and cyclic rail seat bending tests	Aim of investigations was (i) to evaluate the strain, stress, deformation, and load capacity of smart sleepers; (ii) to assess the impact of temperature gradients on sleeper flexural response; (iii) to infer differential ballast settlement using self-sensing sleeper data; (iv) to integrate robust sensors for the acquisition of sleeper information during manufacturing, installation, and operation. The research methods applied: (i) Finite Element Method (FEM) modeling; Discrete Element Method (DEM) modeling. Validation Phases: the validation using field and experimental measurements	Compared to traditional concrete sleepers and smart sleepers with different types of embedded sensors	The integration of supplementary carbon fibers and nanotubes in concrete sleepers significantly improves the mechanical performance and monitoring capabilities. Strain readings indicate non-linear sleeper behavior, and micro-crack initiation can be effectively detected. Pre-tensioned concrete sleepers with embedded sensors show that a temperature-induced curl can add up to 20% to the negative center flexural demand experienced by the sleeper
[43]	FFU (Fiber-Reinforced Foamed Urethane) sleepers and concrete sleepers	FFU sleepers: synthetic material. Concrete sleepers: steel-reinforced concrete	N/A	Not applicable	Not applicable	Not applicable	Numerical simulation using Finite Element Method (FEM). Tests conducted: bending moment distribution and displacement measurements	The aim was to analyze the response of a turnout system with unsupported sleepers via numerical simulations capable of capturing dynamic forces. Research methods: multi-body simulations, dynamic train-turnout interaction analysis, and validation through field measurements	The study compares FFU sleepers with concrete bearers, focusing on their performance in terms of bending moments and displacements under unsupported conditions	The FFU sleepers showed superior performance in reducing bending moment fluctuations and were less sensitive to variations in velocity compared to concrete sleepers. Specifically, FFU sleepers enhanced the turnout performance by providing smoother transitions between turnout panels, with maximum positive bending moments approximately 28% higher than the normal track section, while concrete sleepers showed an 80% increase
[44]	Unknown-type concrete sleeper	Steel-reinforced concrete	Pre-stressed	Yes	Glass-fiber reinforcement in polyamide dowels	N/A	Numerical simulation: non-linear finite element simulation. Experimental validation: thermal infrared sensor measurement and crack opening measurement. Tests executed: thermal cycles with infrared heating	The was to study the effect of temperature on the development of longitudinal cracks in pre-stressed concrete sleepers. Other methods: comparison of the influence of design parameters (concrete aggregates, dowel thickness, and material)	Comparisons between different types of dowel materials (high-density polyethylene, polyamide, and glass fiber-reinforced polyamide) and different types of concrete aggregates (carbonate and siliceous)	The study concluded that sleepers with dowels made of glass fiber-reinforced polyamide, especially those with a higher elastic modulus, showed significant longitudinal cracking under thermal variations. The crack opening increased up to 0.08 mm with a temperature increment of 60 °C in sleepers using carbonate aggregates and thicker dowel geometries
[45]	Unknown-type pre-stressed reinforced concrete beam	Steel-reinforced concrete	pre-stressed	Yes	Fiber-Reinforced Plastic (FRP)	N/A	Numerical simulation (Finite Element Analysis), laboratory experiments, parametric study, investigations included tests for load-displacement, ultimate load capacity, stiffness, and failure mechanisms	The aim was to assess the effects of fiber-reinforced polymer (FRP), beam length, and pre-stressed load on the ultimate loading capacity and stiffness of pre-stressed reinforced concrete beams. The study also aimed to understand the effects of different reinforcement ratios and FRP configurations on strengthened beams. Validation phases involved comparing numerical simulation results with experimental data	Comparisons were made between different beam shapes (rectangular and T-section) and different reinforcement ratios	The inclusion of FRP significantly increases the stiffness and ultimate load capacity of pre-stressed reinforced concrete beams. The best fiber orientation for maximum load capacity is 0 degrees. For example, the average increased maximum load for beams with additional FRP layers is 50% for long rectangular beams, 55% for long T-beams, 150% for short rectangular beams, and 200% for short T-beams
[46]	Unknown-type, concrete sleeper, pre-stressed with CFRP (Carbon Fiber-Reinforced Plastic) tendons	Steel-reinforced concrete	Pre-stressed	Yes	Polypropylene (PP) fibers	2 kg/m^3^	Laboratory experiments, specifically small-scale fire tests. Tests included measuring spalling time, failure time, mode, and the resulting deflections of pre-stressed slabs under fire conditions	The aim was to investigate and improve the spalling behavior of pre-stressed concrete slabs by adding SAP (Superabsorbent Polymers) to the mix. Research methods: Fire tests follow the related ISO standard to assess spalling resistance and deflections. Validation phases included comparison with reference samples without SAP	Comparisons were made between slabs with SAP and reference slabs without SAP	The addition of SAP to HPSCC (High-Performance Self-Compacting Concrete) significantly improved fire spalling resistance. Specimens with SAP showed no spalling, while reference samples did. The combination of SAP and PP fibers allows for maintaining self-compacting properties with lower fiber content, enhancing the overall performance
[47]	Unknown-type concrete sleeper	Steel-reinforced concrete	Pre-stressed	Yes	Fiber-reinforced plastics (FRP)	N/A	Numerical simulation using finite element analysis (FEA). Four-point static loading tests	The aim was to study the behavior of reinforced and pre-stressed concrete structures strengthened by FRP and to develop constitutive models for these materials. Validation: Numerical results were validated against experimental data	Comparisons were made to slabs with and without FRP strengthening	The study concluded that the use of FRP significantly increases the ultimate load capacity of concrete slabs. Specifically, the ultimate load of the slab strengthened with FRP increased by about 84% compared to the slab without FRP
[48]	Unknown-type pre-stressed concrete beams with an I cross-section	Steel-reinforced concrete, CFRP (Carbon Fiber-Reinforced Plastic) strands	Pre-stressed	Yes	Carbon Fiber-Reinforced Plastic (CFRP)	N/A	Laboratory experiments: static tests, including load/deflection curves and fracture behavior analysis. Investigation of fracture behavior, static system analysis, and failure mechanism assessment	The aim was to investigate the behavior of pre-stressed concrete beams reinforced with CFRP strands and compare it with beams pre-stressed with steel strands. Other methods: Comparison of static load tests and fracture mechanisms. Validation phases: Comparison with a basis test sample pre-stressed using steel strands	Comparisons were made to reference sleepers pre-stressed with steel strands	Concrete pre-stressed using CFRP tendons is feasible for statically indeterminate systems. CFRP pre-stressed beams exhibit ductile failure characteristics similar to steel pre-stressed beams, but they do not allow for load transposition due to their pure elastic deformation behavior. This disadvantage can be compensated for by arranging additional strands above the center support
[49]	Unknown-type concrete sleeper (PO). Pre-stressed concrete sleeper with macro synthetic fibers (PF). Pre-stressed concrete sleeper with reduced pre-stressing wires and macro synthetic fibers (PFr)	PO: Steel-reinforced concrete. PF: Steel-reinforced concrete with macro synthetic fibers. PFr: Steel-reinforced concrete with macro synthetic fibers	All considered sleepers (PO, PF, PFr) are pre-stressed	PO: No additional fiber reinforcement. PF: Contains additional fiber reinforcement. PFr: Contains additional fiber reinforcement	PF and PFr: macro synthetic fibers	PF and PFr: 1.0% fiber volume ratio	Life Cycle Cost (LCC) analysis. Comparative study of timber (TS), conventional pre-stressed concrete (PO), and pre-stressed concrete with macro synthetic fibers (PF and PFr)	The aim was to evaluate the life cycle cost of macro synthetic fiber-reinforced concrete (MSFRC) sleepers compared to existing materials in the Australian railway industry. Methods: Financial analysis of acquisition, maintenance, and end-of-life costs. No explicit validation phases were detailed in the document	Comparisons were made between timber sleepers (TS), conventional pre-stressed concrete sleepers (PO), and pre-stressed concrete sleepers with macro synthetic fibers (PF and PFr)	The incorporation of macro synthetic fibers in PF sleepers increased the acquisition costs but provided long-term financial benefits by reducing replacement cycles and maintenance costs. The PF sleeper is recommended for rapid adaptation due to the ease of casting with existing processes

**Table 2:** Compressive strength of cylinder samples.

Designation	Mass	*d*_1_	*d*_2_	*h*	*F*	*f_ci,test_*	*E*	
	[kg]	[mm]	[mm]	[mm]	[kN]	[N/mm^2^]	[N/mm^2^]	
RTV-1	RTV-1a	4.755	104.70	105.46	214.38	709.6	82.1	35,700
	RTV-1b		104.22	105.18	214.26			
RTV-2	RTV-2a	4.709	105.26	105.08	212.30	677.0	78.1	39,400
	RTV-2b		105.60	104.24	212.16			
RT-1	RT-1a	4.796	105.26	105.04	215.32	669.9	77.1	37,700
	RT-1b		105.04	105.34	215.08			
RT-2	RT-2a	4.736	105.32	105.22	212.34	680.7	78.3	38,500
	RT-2b		105.12	105.28	213.36			
STV-1	STV-1a	4.652	105.02	105.24	211.88	672.6	77.4	34,500
	STV-1b		105.06	105.38	211.96			
STV-2	STV-2a	4.664	104.84	105.20	212.82	680.7	78.8	36,700
	STV-2b		104.42	104.94	212.58			
ST-1	ST-1a	4.605	105.14	105.28	210.00	627.8	72.2	34,400
	ST-1b		105.24	105.28	210.06			
ST-2	ST-2a	4.760	105.46	105.18	216.04	652.4	74.9	38,100
	ST-2b		105.46	105.16	215.54			

Note: After the measurements, the *f_ci,test_* (compressive strength of cylinder samples), and *E* (Young modulus for regular and synthetic fiber concrete) were calculated.

**Table 3:** Splitting-tensile strength of cylinder samples.

Designation		Mass	*d*_1_	*d*_2_	*h*	*F*	*f_cti,sp,test_*
		[kg]	[mm]	[mm]	[mm]	[kN]	[N/mm^2^]
RBV-1	RBV-1a	4.954	105.18	105.36	222.12	184.76	5.0
	RBV-1b		104.90	105.74	222.46		
RBV-2	RBV-2a	4.908	105.16	105.28	221.38	175.83	4.8
	RBV-2b		105.12	105.36	221.40		
RB-1	RB-1a	4.781	104.78	105.18	219.10	214.05	5.9
	RB-1b		104.90	105.08	218.18		
RB-2	RB-2a	4.889	105.34	105.18	220.64	199.30	5.5
	RB-2b		105.14	105.26	221.70		
SBV-1	SBV-1a	4.855	105.34	105.18	220.18	190.92	5.2
	SBV-1b		105.30	104.86	220.22		
SBV-2	SBV-2a	4.844	105.28	105.36	219.42	177.51	4.9
	SBV-2b		105.16	105.30	219.12		
SB-1	SB-1a	4.845	105.22	105.22	219.92	163.85	4.5
	SB-1b		104.90	105.26	219.36		
SB-2	SB-2a	4.815	105.26	105.26	219.40	134.83	3.7
	SB-2b		104.88	105.30	220.24		

**Table 4:** Characteristics of the applied hydraulic cylinder.

Manufacturer	Type	Factory Number	Cylinder Power/ Performance	Lifting Height	Cylinder Diameter	Max. Pressure
Hi-Force	HSS504	BK0603	50 tons	102 mm	127 mm	700 bar

**Table 5:** Characteristics of the applied load cell.

Manufacturer	Type	Admissibility Criterion (Accuracy)
MOM-Kaliber	7924 (500 kN)	Precision: 0.10%

**Table 6:** Characteristics of the applied measuring amplifier.

Manufacturer	Type	Channel Number	Sampling Rate	Resolution	Operating Temperature	Accuracy Class
HBM	QuantumX	8	40 kS/s	24 Bit	−20… + 65 °C	0.05%

**Table 7:** Characteristics of the applied measurement data acquisition software.

Manufacturer	Type	Data Collection Speed	Display	Analyze Data during Measurement
HBM	Catman Easy DAQ	12 MS/s; 100 MB/s	Real-time	General scientific calculations Calculations of strength tests

**Table 8:** Characteristics of the applied computer.

**Manufacturer**	**Type**	**Processor**	**Memory**	**Operating System**
LENOVO	20LX-S1GK00	Intel^®^ Core™ i5-8250U CPU @ 1.60 GHz 1.80 GHz	8.00 GB	64 bit

**Table 9:** Test Specifications. Requirements for MABA L4-type sleeper.

Type of Sleeper	Cross-Section under the Rail					In the Middle (Middle of the Support)			
	Length [cm]	*M_dr_* [kNm]	*M_dr_*_28_ [kNm]	*F_dr_*_28_ [kN]	Height [mm]	*M_dcn_* [kNm]	*M_dcn_*_28_ [kNm]	*F_dcn_*_28_ [kN]	Height [mm]
MABA L4 *Q* ≤ 250 kN *V* ≤ 200 km/h	260.00	17.00	21.30	170.00	215.00	−10.6	−13.3	38.00	185.00

**Table 10:** Experimental concrete properties in compression and tension.

Designation	*f_c_*	*E*	*f_t_*
	[N/mm^2^]	[N/mm^2^]	[N/mm^2^]
RTV-1	82.1	35,700	5.0
RTV-2	78.1	39,400	4.8
RT-1	77.1	37,700	5.9
RT-2	78.3	38,500	5.5
STV-1	77.4	34,500	5.2
STV-2	78.8	36,700	4.9
ST-1	72.2	34,400	4.5
ST-2	74.9	38,100	3.7

**Table 11:** CDP input data for concrete (the data were mainly adopted from [19]).

Mix Type	Dilation Angle	Eccentricity	*f_b_*_0_ */f_c_*_0_	*K*	Viscosity
Regular concrete	35	0.2	1.16	0.667	0.0079
Fiber-reinforced concrete	37	0.2	1.16	0.667	0.005

**Table 12:** Measurement results of the bending tests related to the examination of the cross-section under the rail in the installation position.

Load (Force) Values [kN]	Designation of Test Specimens					
	R1_1	R1_2	avg.	S1_1	S1_2	avg.
*F_ro_*	136.0	136.0	136.0	136.0	136.0	136.0
*F_rr_*	170.0	180.0	175.0	160.0	168.0	164.0
*F_r_*_0.10_	220.0	210.0	215.0	240.0	240.0	240.0
*F_r_*_0.05_	320.0	320.0	320.0	280.0	270.0	275.0
*F_rB_*	438.52	434.25	436.39	441.56	426.10	433.83

Note: (R: regular (reference); S: synthetic fiber-reinforced), e.g., The examination of the cross-sections under the rail R1_1 and R1_2 took place on the same sleeper (on both ends), similarly for fiber-reinforced S1_1 and S1_2.

**Table 13:** Measurement results of the bending tests related to the examination of the central cross-section of the sleeper in an inverted position for a negative moment.

Load (Force) Values [kN]	Designation of Test Specimens	
	Rf_1	Sf_1
*F_con_*	30.0	30.0
*F_crn_*	45.0	45.0
*F_c_*_0.10*n*_	70.0	60.0
*F_c_*_0.05*n*_	-	-
*F_cBn_*	95.2	97.7

Note: (R: regular (reference); S: synthetic fiber-reinforced).

**Table 14:** Measurement results of the bending tests related to the examination of the central cross-section of the sleeper in the normal position for a positive moment.

Load (Force) Values [kN]	Designation of Test Specimens	
	Rno_2	Sno_1
*F_co_*	30.0	30.0
*F_cr_*	55.0	60.0
*F_c_*_0.10_	60.0	80.0
*F_c_*_0.05_	-	-
*F_cB_*	106.0	111.6

Note: (R: regular (reference); S: synthetic fiber-reinforced).

**Table 15:** Calibrated deflection values (the “???” sign means that the GOM software cannot measure accurately the displacements at the pre-given, pre-chosen points due to the tear of the paint on the surface).

Case	*P* [kN]	Vertical Deflection Results
S (Exp.)	136	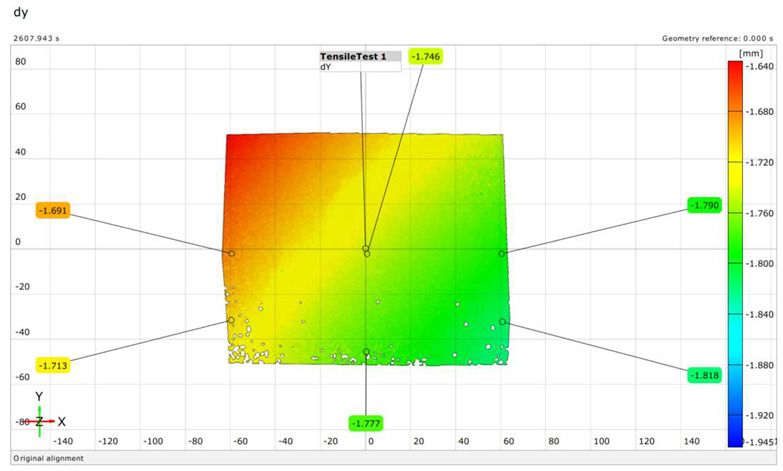
S (Num.)	136	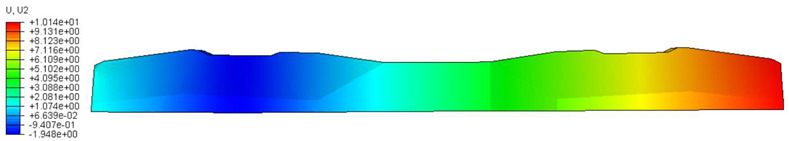
R_f_ (Exp.)	93	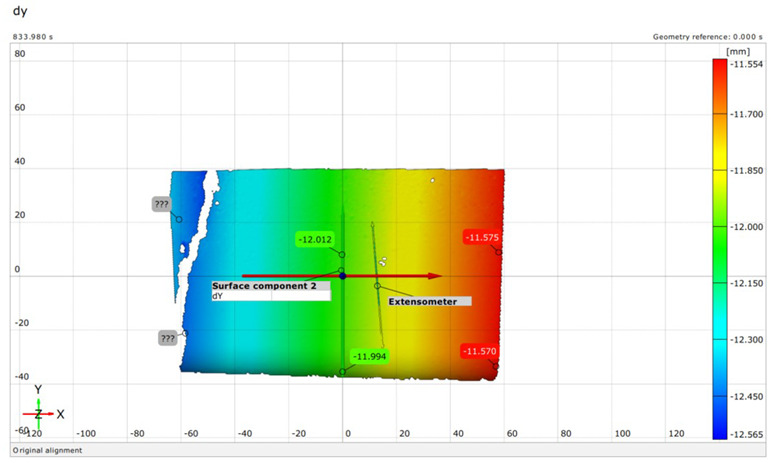
R_f_ (Num.)	93	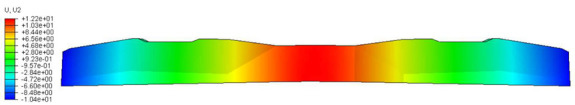
S_f_ (Exp.)	97	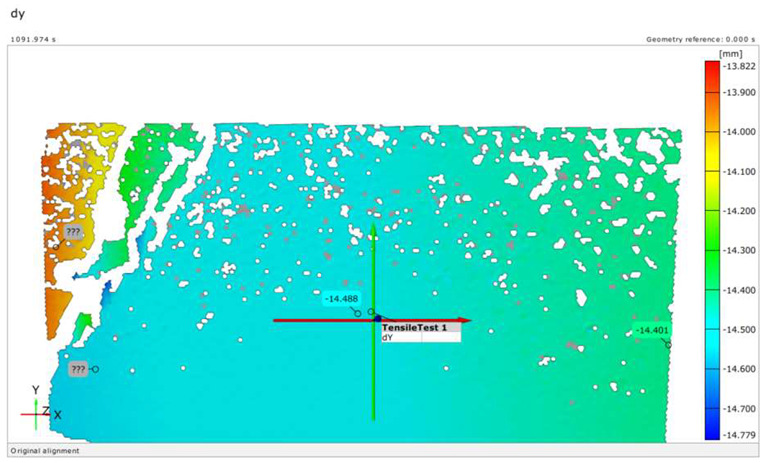
S_f_ (Num.)	97	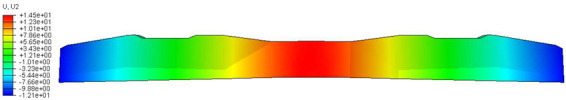
R_no_ (Exp.)	95	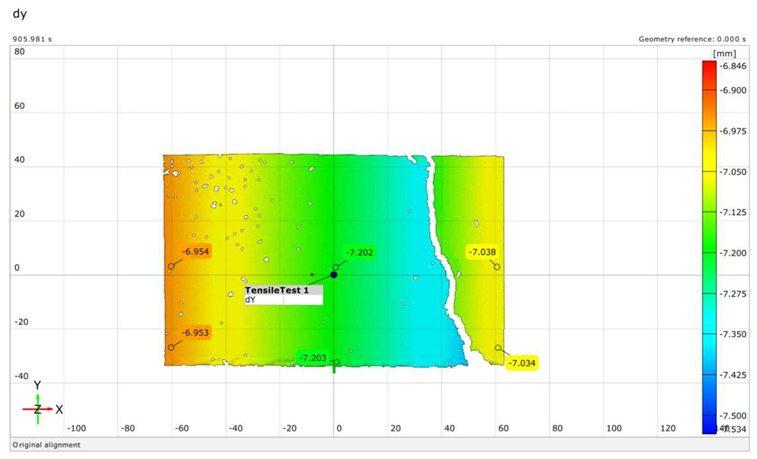
R_no_ (Num.)	95	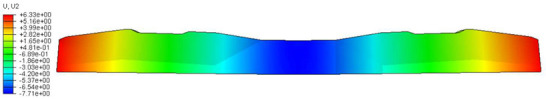

**Table 16:** Numerical results of the calibrated sleepers—Concrete part.

Case	Max. Load (Force) *P* [kN]	Max. Deflection ∆ [mm]	Section	Tension Damage Coefficient (*d_t_*) 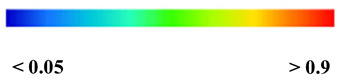
R	431.66	8.5	-	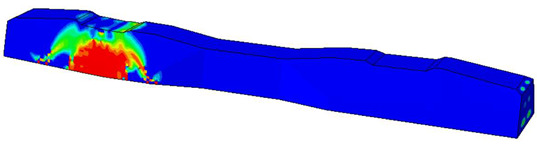
			A-A	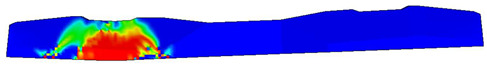
			C-C	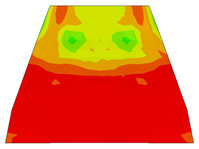
			D-D	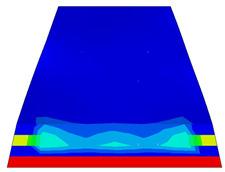
S	449.89	8	-	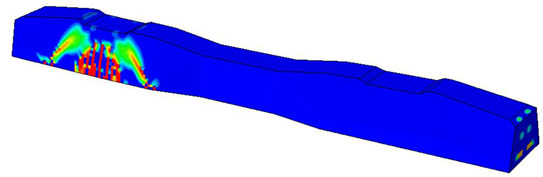
			A-A	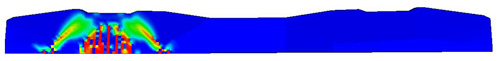
			C-C	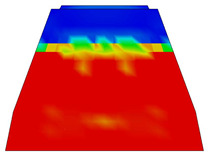
			D-D	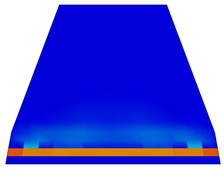
R_f_	93.61	12.46	-	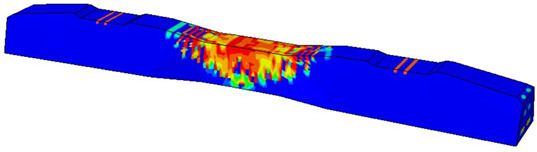
			A-A	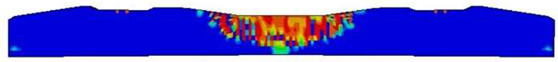
			B-B	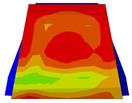
			C-C	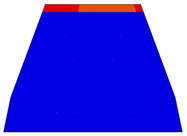
S_f_	96.97	14.79	-	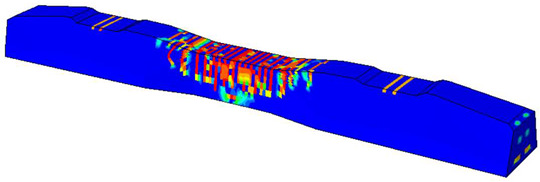
			A-A	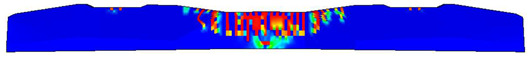
			B-B	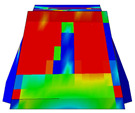
			C-C	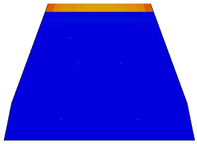
R_no_	97.5	7.59	-	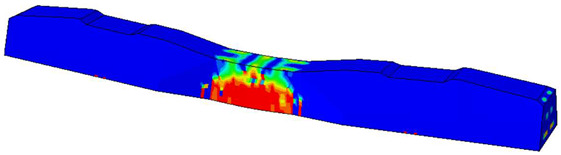
			A-A	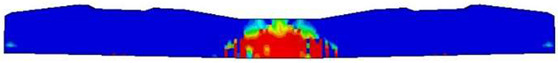
			B-B	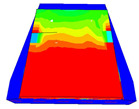
			C-C	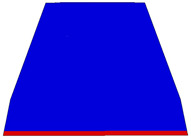
S_no_	102.6	7.91	-	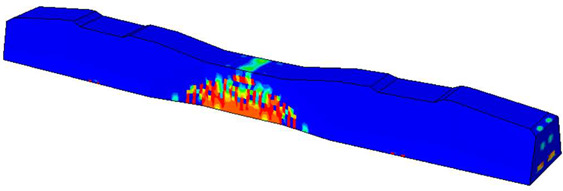
			A-A	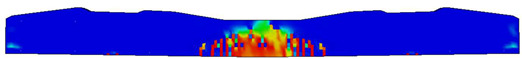
			B-B	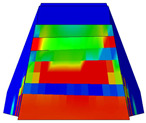
			C-C	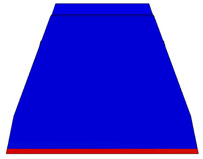

**Table 17:** Numerical results of the calibrated sleepers—Steel part.

Case	Section	Steel Stress Intensity (*σ*/*σ_y_*) 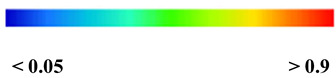
R	-	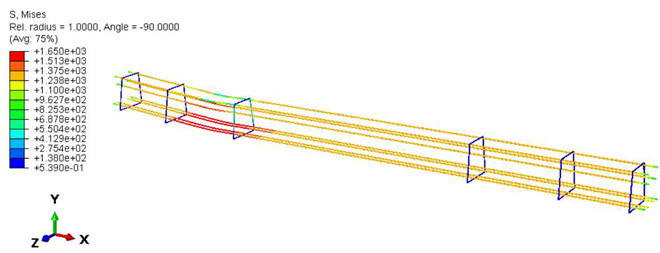
	A-A	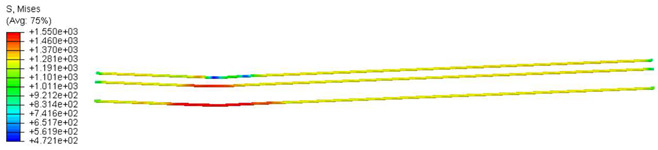
	C-C	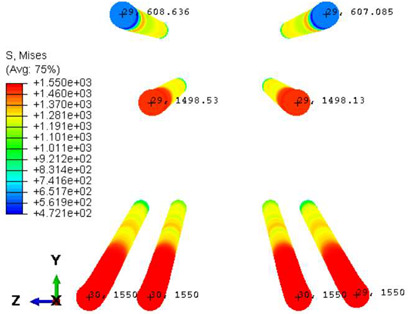
S	-	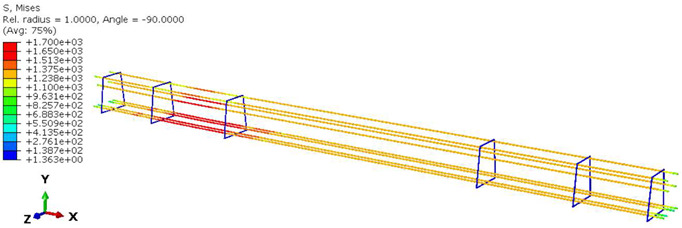
	A-A	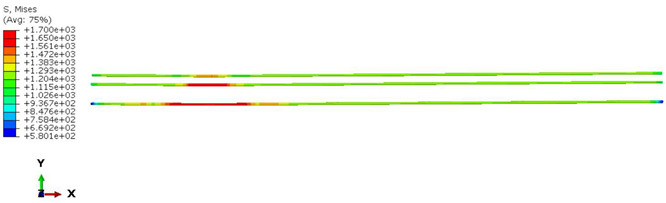
	C-C	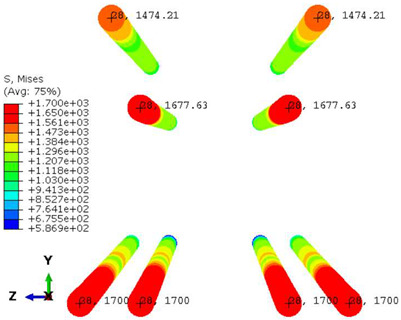
R_f_	-	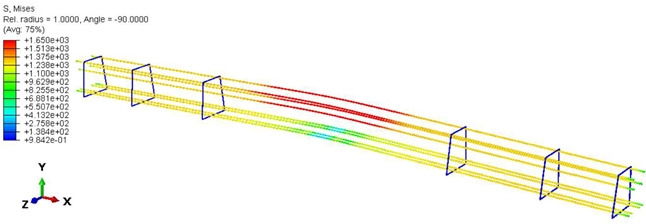
	A-A	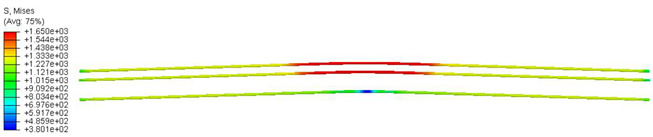
	B-B	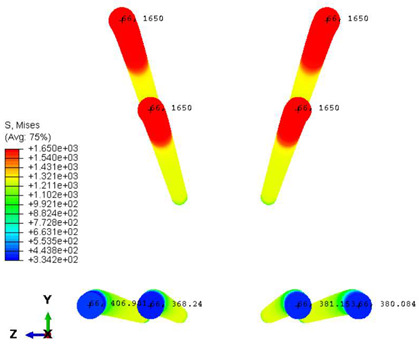
S_f_	-	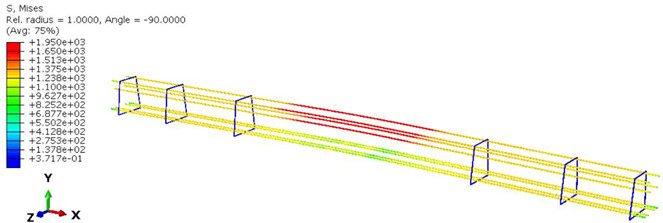
	A-A	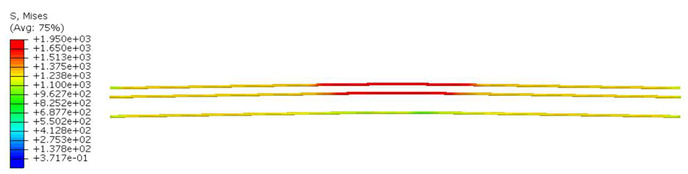
	B-B	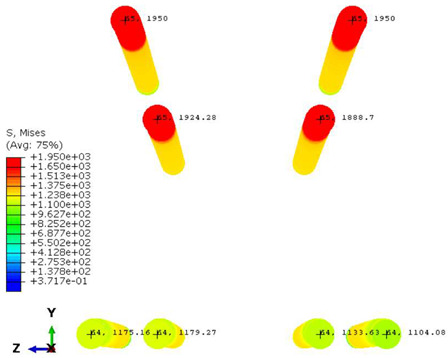
R_no_	-	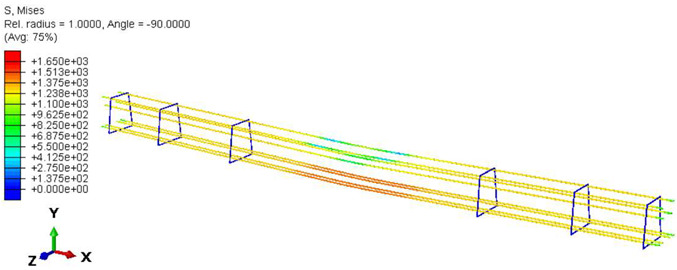
	A-A	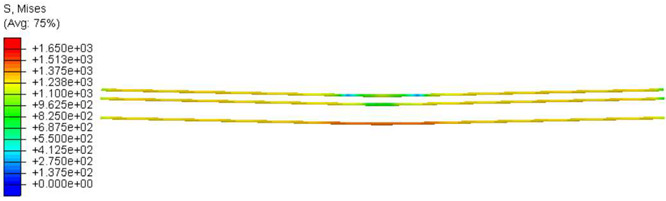
	B-B	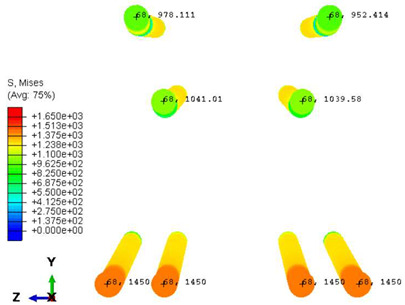
S_no_	-	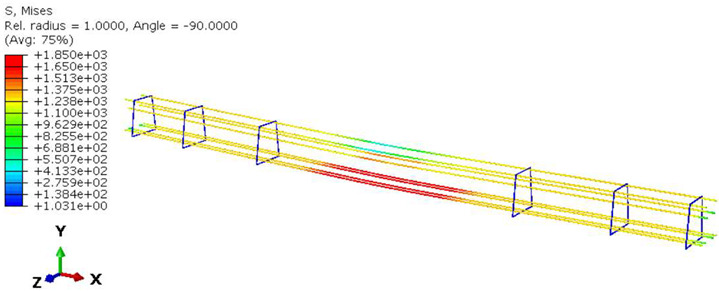
	A-A	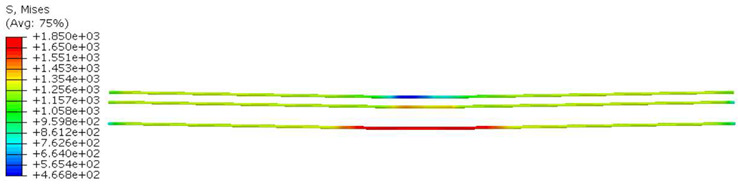
	B-B	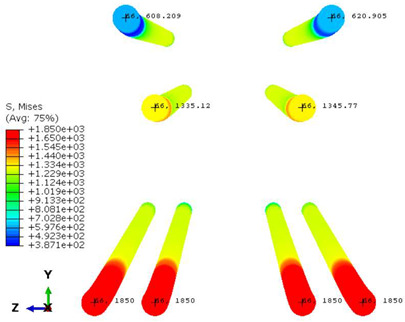

## Data Availability

Data are contained within the article.

## Abbreviations

3PBT	Three-Point Bending Test
ABAQUS	A sophisticated software based on finite element method calculation which is mainly used for research and development
ANSYS	A sophisticated software based on finite element method calculation which is mainly used for research and development
CDM	Concrete Damage Plasticity Constitutive Model
CDP	Concrete Damage Plasticity
CF	Carbon Fibers
CFRP	Carbon Fiber Reinforced Polymer
CFRPC	Carbon Fiber Reinforced Polymer Concrete
CNF	Carbon Nanofibers
CNT	Carbon Nanotubes
DEM	Discrete Element Method
DIC	Digital Image Correlation
DOF	Degree of Freedom
FE	Finite Element
FEM	Finite Element Method or Finite Element Modeling
FFU	Fiber-Reinforced Foamed Urethane
FRF	Frequency Response Function
FRP	Fiber Reinforced Plastic or Fiber Reinforced Polymer
FRP-OF	Fiber Reinforced Polymer-Optical Fiber
GFRP	Glass Fiber Reinforced Polymer
GGBFS	Ground Granulated Blast Furnace Slag
HPSCC	High-Performance Self-Compacting Concrete
L-CFRPU	Laminated Carbon Fiber Reinforced Polyurethane
MSFRC	Macro-Synthetic Fiber Reinforced Concrete
PC	Polymer Concrete
PGRS	Pre-stressed Geopolymer Railway Sleepers
PPF	Polypropylene Fiber
R	Regular (Reference)
RB	Reference, splitting-tensile strength after storage in air [N/mm^2^]
RBV	Reference, splitting-tensile strength after storage in water [N/mm^2^]
RC	Reinforced Concrete
RCPT	Rapid Chloride Penetration Test
RPC	Reinforced Polymer Concrete
RT	Reference, compressive strength after storage in air [N/mm^2^]
RTV	Reference, compressive strength after storage in water [N/mm^2^]
S	Synthetic fiber-reinforced
SAP	Superabsorbent Polymers
SB	Synthetic fiber-reinforced, splitting-tensile strength after storage in air [N/mm^2^]
SBV	Synthetic fiber-reinforced, splitting-tensile strength after storage in water [N/mm^2^]
SEM	Scanning Electron Microscope
SFRGRS	Steel Fiber Reinforced Geopolymer Railway Sleepers
SHM	Structural Health Monitoring
ST	Synthetic fiber-reinforced, compressive strength after storage in air [N/mm^2^]
STV	Synthetic fiber-reinforced, compressive strength after storage in water [N/mm^2^]
TAL	tons axle load
UHPC	Ultra-High Performance Concrete
UHP-FRC	Ultra-High Performance Fiber-Reinforced Concrete
VB	Vee-Bee Consistometer
XRD	X-ray Diffraction

## Nomenclature

*ν*	Poisson’s ratio [–]
*σ*	Normal stress [MPa] or [Pa]
*σ_c_*	Compression (compressive) stress [MPa] or [Pa]
*σ_t_*	Tensile stress [MPa] or [Pa]
${\bar{\sigma }}_{Cij}$	Actual internal compression force [MPa] or [Pa]
${\hat{\sigma }}_{ij}$	Internal force tensor value [MPa] or [Pa]
${\bar{\sigma }}_{ij}$	Actual internal force [MPa] or [Pa]
${\bar{\sigma }}_{tij}$	Actual internal tension force [MPa] or [Pa]
∆	Vertical deflection of the sleeper in ABAQUS modeling [mm]
*ε_ij_*	General strain tensor value [–]
*ε_ij_^el^*	Elastic part of the general strain tensor value [–]
*ε_ij_^pl^*	Plastic part of the general strain tensor value [–]
*ε_t_*	Tensile strain [–]
$\epsilon_{c}^{el}$	Elastic strain in compression [–]
$\epsilon_{c}^{pl,h}$	Plastic strain in compression [–]
$\epsilon_{t}^{el}$	Elastic strain in tension [–]
$\epsilon_{t}^{pl,h}$	Plastic strain in tension [–]
*d*	Thickness of the sleeper under the rail foot’s center [m]
*d′*	Scalar stiffness degradation variable [–]
*d_c_*	Compression damage variable [–]
*d_t_*	Tension damage variable [–]
$D_{0}^{el}$	Initial (undamaged) elastic stiffness of the material [kN/mm]
$D_{ijkl}^{el}$	Degraded elastic stiffness [kN/mm]
*E*	Young’s moduli [N/mm^2^]
*E*_0_	Material initial (undamaged) Young’s modulus
*f*_*b*0_	Equibiaxial compressive strength of concrete [MPa]
*f*_*c*0_	Uniaxial compressive strength of concrete [MPa]
*F*_*c*0_	Value given by the manufacturer based on the required load-bearing capacity [kN]
*F*_*c*0.05_	Force value causing a crack width of 0.05 mm remaining after unloading [kN]
*F*_*c*0.10_	The force value causing a crack width of 0.1 mm [kN]
*F_cB_*	A force value that can no longer be increased (causing breakage) [kN]
*f_ci,test_*	Compressive strength of cylinder samples, [N/mm^2^]
*F_con_*	Value given by the manufacturer based on the required load-bearing capacity [kN]
*F_cr_*	The force value that causes the first crack [kN]
*f_cti,sp,test_*	Splitting-tensile strength of cylinders [N/mm^2^]
*F*_*r*0.05_	Force value causing a crack width of 0.05 mm remaining after unloading [kN]
*F*_*r*0.10_	The force value causing a crack width of 0.1 mm [kN]
*F_rB_*	A force value that can no longer be increased (causing breakage) [kN]
*F_ro_*	Value given by the manufacturer based on the required load-bearing capacity [kN]
*F_rr_*	The force value that causes the first crack [kN]
*K*	Ratio of the second stress invariant on the tensile meridian in ABAQUS modeling [–]
*L_c_*	Design distance between center lines of the rail seat for the test arrangement of mid-span loading based on EN 13230-2 [56] [m]
*L_r_*	Design distance between the articulated supports center lines for the test arrangement at the rail seat section based on EN 13230-2 [56] [m]
*M*	Mean value of bending moment [kNm]
*M_d_*	Design value of bending moment [kNm]
*M_dc_*	Value of the bending moment causing cracking related to the sleeper’s center [kNm]
*M_dr_*	Value of the bending moment causing cracking under the rail foot [kNm]
*P*	Vertical load acting on the sleeper in ABAQUS modeling [kN]
